# Targeting p53 pathways: mechanisms, structures and advances in therapy

**DOI:** 10.1038/s41392-023-01347-1

**Published:** 2023-03-01

**Authors:** Haolan Wang, Ming Guo, Hudie Wei, Yongheng Chen

**Affiliations:** Department of Oncology, NHC Key Laboratory of Cancer Proteomics, Laboratory of Structural Biology, National Clinical Research Center for Geriatric Disorders, Xiangya Hospital, Central South University, Changsha, Hunan 410008 China

**Keywords:** Drug development, Drug development, Structural biology

## Abstract

The *TP53* tumor suppressor is the most frequently altered gene in human cancers, and has been a major focus of oncology research. The p53 protein is a transcription factor that can activate the expression of multiple target genes and plays critical roles in regulating cell cycle, apoptosis, and genomic stability, and is widely regarded as the “guardian of the genome”. Accumulating evidence has shown that p53 also regulates cell metabolism, ferroptosis, tumor microenvironment, autophagy and so on, all of which contribute to tumor suppression. Mutations in *TP53* not only impair its tumor suppressor function, but also confer oncogenic properties to p53 mutants. Since p53 is mutated and inactivated in most malignant tumors, it has been a very attractive target for developing new anti-cancer drugs. However, until recently, p53 was considered an “undruggable” target and little progress has been made with p53-targeted therapies. Here, we provide a systematic review of the diverse molecular mechanisms of the p53 signaling pathway and how *TP53* mutations impact tumor progression. We also discuss key structural features of the p53 protein and its inactivation by oncogenic mutations. In addition, we review the efforts that have been made in p53-targeted therapies, and discuss the challenges that have been encountered in clinical development.

## Introduction

The tumor suppressor gene *TP53* is the most frequently mutated gene in human tumors.^1,2^ The process of tumor development is strongly related to the dysfunctions caused by *TP53* mutations.^3,4^ p53 protein functions primarily as a transcription factor, which regulates a wide variety of pathways, such as cell cycle arrest, DNA repair, cell apoptosis, autophagy, and metabolism,^1,5,6^ and determines whether cells die under stress conditions. Over the years, a growing number of studies have revealed the complexity and connectivity of the p53 pathway and by extension its role in metabolic homeostasis, immune microenvironment, stem cell biology and so on. However, mutant p53 can alter DNA-specific binding, disrupt the spatial conformation of the protein and thermostability of the protein, and result in dysfunction of p53 activity.^7–10^

The high frequency of *TP53* mutations in tumors and its intrinsic tumor suppressor function make it a highly promising target for tumor therapy. However, the specificity of the p53 structure,^11,12^ the smooth surface without an ideal drug-binding pocket,^13^ and the difficulty to restore p53 function, have stalled drug research against p53 for decades. Nevertheless, researchers still believe that this difficult-to-drug target can be tackled, and have made some progress in recent years. In this review, we aim to provide a comprehensive summary of the biological function of p53, p53 signaling pathway, the structural features of p53 protein, as well as the advances in p53-targeted therapies.

### The discovery history of p53

**Fig. 1:**
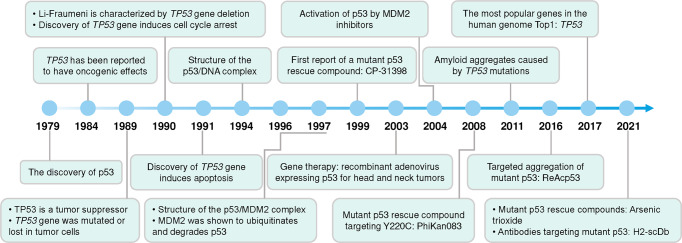
Timeline of some major advances in p53 research

The *TP53* gene is located on the short arm of chromosome 17 (17p13.1) and encodes a protein with 393 amino acid residues. p53 was initially identified as a host protein that bound to simian virus 40 large T antigen in virally transformed cells,^14^ and was named p53 in 1979 because its molecular weight was shown to be approximately 53 kilodalton (kDa) in SDS polyacrylamide gel electrophoresis analysis^15^ (Fig. 1). The actual molecular weight of p53 is 43.7 kDa, due to the large number of proline residues in the protein that slowed down its migration on SDS polyacrylamide gel electrophoresis. *TP53* was initially thought to be an oncogene, and high levels of p53 conferred significant tumorigenic potential to rat embryonic fibroblasts.^16,17^ Subsequent studies have led to a change in the recognition of *TP53*. The initial p53 cDNA was synthesized using mRNA from tumor cells as a template, and the p53 cDNA subsequently obtained from normal cells did not transform the cells, but rather inhibited tumor cell growth.^18^ In tumor cells the *TP53* gene is often mutated or lost due to chromosome 17 deletion, whereas in normal cells the gene is intact.^19,20^ When a missense mutation in *TP53* occurs, the obtained p53 protein promotes tumorigenic transformation.^21,22^ *TP53* knockout mice have a high probability of developing tumors.^23^ Overexpression of the wild-type *TP53* gene in cells effectively suppressed the transforming effects exerted on cells by those oncogenes, such as the MYC gene and RAS gene.^22,24^ This series of studies overturned the established paradigm of *TP53*, which has since become one of the most studied tumor suppressor genes.

### The p53 pathway

p53 is a transcription factor that is distributed in the nucleus and cytoplasm, binds specifically to DNA, and regulates a diversity of genes.^25,26^ Under normal conditions, cellular p53 protein levels are very low owing to strict control by its negative regulators MDM2 and MDMX, which promote p53 degradation through ubiquitination.^27,28^ When cells are exposed to internal and external stresses, including DNA damage, hypoxia, nutrient deprivation, and cancer cell risk, p53 ubiquitination is inhibited, triggering a rapid increase in intracellular p53 protein levels. Accumulated p53 is activated and stabilized by posttranslational modifications, including phosphorylation, acetylation and methylation.^29–32^ Stabilized p53 forms tetramers in the nucleus, binds to target DNA and regulates gene transcription, leading to alterations in downstream signaling pathways.^33–37^

**Fig. 2:**
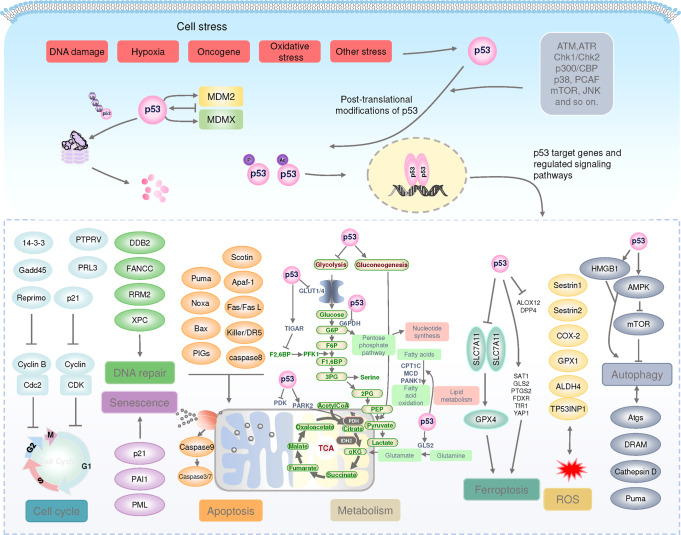
The p53 pathway. Under normal conditions, p53 protein levels are tightly regulated by MDM2/X, which together ubiquitinate p53, leading to proteasomal degradation of p53. Under stress conditions, p53 is activated and stabilized by post-translational modifications. Stabilized p53 forms tetramers in the nucleus, binds to target DNA, regulates gene transcription, and controls many different biological processes. ALDH4 aldehyde dehydrogenase family 4 member A1, ALOX12 arachidonate-12-lipoxygenase, AMPK 5′-AMP-activated protein kinase, APAF1 apoptotic protease-activating factor 1, Atgs autophagy-related genes, ATM ataxia-telangiectasia mutated proteins, ATR ataxia telangiectasia and Rad3-related, BAX apoptosis regulator BAX, CDC cell division cycle, CDK cyclin-dependent kinase, COX-2 cyclooxygenase-2 (also known as PTGS2), CPT1C carnitine palmitoyltransferase 1C, Cyt C Cytochrome C, DDB2 damage specific DNA binding protein 1, DPP4 dipeptidyl peptidase-4, DRAM damage regulated autophagy modulator 2, FANCC fanconi anemia group C protein, FDXR ferredoxin reductase, GADD45 growth arrest and DNA damage-inducible 45, GLS2 glutaminase 2, GLUT glucose transporter type, GPX glutathione peroxidase, G6PDH glucose‑6‑phosphate (G-6‑P) dehydrogenase, HMGB1 high-mobility group box-1, MCD malonyl-coenzyme A decarboxylase, mTOR mammalian target of rapamycin, NOXA superoxide-generating NADPH oxidase, PAI1 plasminogen activator inhibitor 1, PANK1 pantothenate kinase 1, PDK PDH kinase, PIGs p53-induced gene, PML promyelocytic leukemia protein, PRL3 phosphatase of regenerating liver-3, PTPRV protein tyrosine phosphatase receptor type V, PUMA Bcl-2-binding component 3, ROS reactive oxygen species, RRM2 ribonucleoside-diphosphate reductase subunit M2, SAT1 spermine N1-acetyltransferase 1, SLC7A11 solute carrier family7 member11, TFR1 transferrin receptor 1, TIGAR TP53-induced glycolysis and apoptosis regulator, TP53INP1 tumor protein p53 inducible nuclear protein 1, XPC xeroderma pigmentosum group C protein, YAP1 yes-associated protein 1

In response to cellular stress, p53 prevents the differentiation of cells with mutated or damaged DNA and terminates cellular processes by transcriptionally activating various genes involved in apoptosis and cell cycle,^38,39^ which contributes significantly to its tumor suppressor function and is the most studied.^33–37,40–44^ Meanwhile, a series of studies have shown that p53 also controls a number of “non-classical” pathways (Fig. 2), including metabolic homeostasis, ferroptosis, stem cell differentiation, autophagy, senescence, tumor microenvironment and so on.^45–50^

#### The biological function of p53 in cancer

p53 controls a wide range of signaling networks. There is no simple and clear answer to the question of exactly how, when, and what p53 does. Nevertheless, what is clear is that p53 has a very flexible and versatile response, with an integrated response to environmental perturbations determining cell death or maintaining cellular homeostasis. p53 serves as a linkage point for multiple cellular signaling pathways, harmoniously and delicately regulating various biological functions through transcriptional regulation and protein-protein interactions.

##### Genomic stability

p53 is considered as the guardian of the genome. It plays an important role in maintaining genomic stability. When DNA is damaged, p53 protects the genome by coordinating multiple DNA damage response mechanisms.^51^ The p53 protein activates the expression of DNA repair proteins DDB2 and XPC.^52^ The interaction of these proteins with effector proteins may lead to various cell fates, such as apoptosis, senescence or tumorigenesis.^53,54^

The gene encoding p21 (CDKN1A) is the first transcriptional target identified for p53.^55^ p21 is a potent inhibitor of binding to cyclin-dependent kinases (CDKs), which inhibits cell cycle proteins and further inhibits phosphorylation of Rb by the cyclin D1-CDK4, cyclin D2-CDK4 and cyclin E-CDK2 complexes.^56–58^ Hypophosphorylated Rb forms a complex with the E2F transcription factor, which inhibits the transcriptional activity of E2F and leads to G1 phase block.^40,59^ PTPRV, which encodes a transmembrane tyrosine phosphatase, and the phosphatase of regenerating liver-3 are both genes involved in p53-induced G1 phase block.^60,61^

In addition, p53 represses CDKs and cyclin B, which are required for entry into mitosis and are involved in G2/M phase block.^62,63^ p53 induces transcription of 14-3-3 sigma and represses the cell cycle protein-dependent kinase Cdc2.^64^ Gadd45 is a member of the growth arrest and DNA damage gene family. p53 regulates the transcription of Gadd45, disrupts the cyclin B1/Cdc2 complex, and further blocks the G2 phase.^63,65^ Reprimo is also involved in p53-induced cell cycle arrest in the G2 phase.^66^ These modulations of p53 reduce the risk of gene mutation and prevent the activation of oncogenes.

On the other hand, p53 can also induce apoptosis in cells with DNA damage.^67^ p53 causes apoptosis through transcriptional activation of the expression of the pro-apoptotic genes, such as Puma, Bax and Noxa.^68–70^ p53 is also thought to regulate mitochondrial apoptosis through a transcription-independent pathway.^33^ p53 binds physically to anti-apoptotic proteins (Bcl-2, Bcl-xL and Mcl-1), thereby indirectly inducing apoptosis.^71^ p53 directly activates the pro-apoptotic protein Bak or disrupts the Mcl-1 and Bak complex, releasing Bak and initiating apoptosis.^37,72^ In addition, p53-driven miR34a expression may sensitize cells to apoptotic stimuli by decreasing Bcl-2 levels.^73,74^ In the exogenous apoptotic pathway, p53 induces the expression of the death receptors Fas/Fas ligand and KILLER/DR5 located on the cell membrane, which activates caspase 8 and leads to apoptosis.^75,76^ p53 also drives the expression of various genes that may have pro-survival or pro-apoptotic functions,^77,78^ which also contribute to the maintenance of genomic stability.

Retrotransposons are replicated and inserted into new genomic sites by reverse transcription of RNA intermediates, which allows them to increase the copy number or gene mutations in the host genome.^79^ Disinhibition of retrotransposons has been reported to be closely associated with human tumorigenesis.^80^ p53 binds to the promoter region of the retrotransposon element LINE1 and prevents the expression of transposon sequences.^81^ When p53 is absent, cells overexpressing synthetic retrotransposon genes are able to avoid apoptosis.^82^ Genomic instability caused by deletion or mutation of p53 may accumulate more oncogenes and promote tumorigenesis, proliferation, metastasis and drug resistance. Functional inactivation of p53 not only contributes to genomic mutation and copy number increase, but also maintains the survival of cells carrying faulty genetic information.

##### Senescence

Senescence is a permanent cell cycle arrest. p53-mediated senescence is closely related to its tumor suppressive effects. DNA damage triggers senescence, a process often referred to as stress-induced premature senescence.^83^ Various internal or external stressors trigger the DNA damage response pathway, activating the p53 and/or p16INK4A pathways.^84^ p16INK4A inactivates Cdk4/6, phosphorylates Rb accumulation and inactivates E2F transcription, leading to cell cycle arrest or senescence.^85^ Alternatively, when UV-induced DNA damage occurs, ATM/ATR activates Chk1/Chk2 kinase, which further activates p53 and p21^CIP1^, leading to G1 arrest or senescence.^86^ In addition, p21^CIP1^ protein levels may lead to inhibition of CDK4/6 activity, resulting in G1 arrest or senescence.^87^ In addition, p53 directly induces senescence by stabilizing fibrinogen activator inhibitor-1, a marker of senescent cells.^88^ p53 also activates the transcription of genes that encode promyelocytic leukemia protein, leading to cellular senescence.^89^

##### Metabolic homeostasis

Tumor cells require large amounts of biological raw materials and energy to achieve their rapid and sustained growth. The Warburg effect,^90^ which was first proposed, states that tumor cells metabolize glucose differently than normal cells, as evidenced by enhanced glycolysis and increased lactate production.^91^ p53 regulation of the glycolytic pathway helps maintain the homeostasis of cellular metabolism and thus acts as a tumor suppressor. p53 can transcribe target genes required for oxidative phosphorylation, such as SCO2,^92^ or genes that inhibit glycolysis, such as TIGAR and Parkin.^93,94^ p53 binds to G6PDH, the rate-limiting enzyme of the pentose phosphate pathway, to further inhibit the activated pentose phosphate pathway in tumor cells.^95,96^ p53 inhibits glucose uptake and glycolysis by suppressing the expression and translocation of glucose transporter proteins such as GLUT1 and GLUT4.^97^ Glycolysis and gluconeogenesis can be considered reversible processes to some extent. p53 inhibition of glycolysis promotes the process of gluconeogenesis.^98^ Since tumor cells are highly dependent on glycolysis and the Warburg effect for proliferation and invasion, p53 inhibition of glycolysis tends to impede cancer cell growth.^97,99,100^

Cancer cells may indeed activate different metabolic pathways under different environmental conditions. Mutant p53 activates the Warburg effect by promoting the translocation of the GLUT1 to the plasma membrane, thereby enhancing tumor metabolism.^101^ Mutant 53 binds and activates PGC-1α, a major regulator of oxidative phosphorylation, enhancing mitochondrial function and promoting cancer metastasis.^102,103^ These studies suggest that mutant p53 may confer metabolic plasticity to cancer cells, thereby promote their adaptation to metabolic stress and increase their potential for proliferation and metastasis.

Since tumor cells need to accumulate or synthesize lipids to promote growth and proliferation,^104^ p53 promotes lipolysis leading to tumor suppression.^46^ The mevalonate pathway is responsible for the biosynthesis of cholesterol and nonsteroidal isoprenoids, and SREBP2 is a major transcriptional regulator of this pathway.^105^ p53 blocks the activation of SREBP2 by transcriptionally inducing the ABCA1 cholesterol transporter gene.^105^ p53 downregulates the expression of USP19 and SOAT1 to inhibit cholesterol esterification.^106^ p53 also promotes fatty acid oxidation by activating the expression of CPT1C, MCD and PANK1.^107^

Ammonia is a prevalent product of cellular metabolism. Tumor cells produce large amounts of ammonia during amino acid metabolism, and this ammonia can serve as a nitrogen source for tumor growth.^108,109^ p53 regulates ammonia content in tumor cells through the urea cycle. p53 regulates ammonia content in tumor cells by inhibiting the expression of three key enzyme genes in the urea cycle of tumor cells, CPS1, OTC and ARG1, which ultimately inhibits tumor growth.^110^

p53 is also involved in regulating the metabolism of tumor cells along with other metabolic signaling pathways. Increased levels of reactive oxygen species in tumor cells have a dual effect, both by promoting the acquisition of a tumor phenotype and by activating ROS-dependent death signals to kill tumor cells.^111,112^ The regulation of ROS by p53 also has a dual function. ROS act as an upstream signal to trigger the activation of p53, and p53 transcribes the expression of multiple antioxidant genes, such as GPX1 and manganese superoxide dismutase, to support tumor cell growth or death.^113–115^ ROS can also act as a downstream factor of p53 to drive tumor cell death through apoptosis and ferroptosis.^45,116^ p53 plays a dual role in inhibiting and promoting the tricarboxylic acid cycle and oxidative phosphorylation.^46,117,118^ p53 is also involved in regulating cellular redox reactions and mediating cancer cell death.^46,119,120^ In addition, p53 is involved in the regulation of lipid, amino acid and nucleotide metabolism.^46,121,122^

##### Ferroptosis

Ferroptosis is a form of regulated cell death initiated notably by severe lipid peroxidation.^123–125^ p53 was reported to inhibit cystine uptake and promote ferroptosis by transcriptional repression the expression of SLC7A11 which is a key component of the cystine-glutamate antiporter.^45,126,127^ p53 expression further enhances the ability of GPX4 to antagonize ferroptosis by increasing the biosynthesis of GSH.^127^ p53 also regulates SLC7A11 expression in a non-transcriptional manner. p53(3KR) is an acetylation-deficient mutant that fails to induce cell cycle arrest, apoptosis and senescence,^128^ but retains the ability to regulate SLC7A11 expression.^45^ Another mutant of p53(4KR) lost the ability to regulate SLC7A11 expression.^126^ This also suggests the importance of acetylation of p53 for the regulation of ferroptosis. H2Bub1 activates SLC7A11 expression. p53 inhibits the level of H2Bub1 by promoting nuclear translocation of the deubiquitinase USP7, which suppresses the expression of SLC7A11 and induces ferroptosis.^129^

p53 can also activate the expression of SAT1, a rate-limiting enzyme in polyamine catabolism, thereby inducing lipid peroxidation and ferroptosis upon ROS stress.^130^ SAT1-induced ferroptosis is involved in elevated expression levels of the lipoxygenase ALOX15.^130^ ALOX12 was also reported to be required for p53-mediated ferroptosis.^131^ Moreover, p53 promotes ferroptosis through modulation of GLS2, PTGS2, FDXR and noncoding RNAs.^132^ On the other hand, interaction of p53 with DPP4 promotes nuclear accumulation of DPP4 and blocks plasma-membrane-associated DPP4-dependent lipid peroxidation, thus limiting ferroptosis.^133^ The p53-p21 axis may contribute to the inhibition of cysteine deprivation-induced ferroptosis,^134^ whereas it has also been reported that p21 restricts the progression of ferroptosis in a p53-independent way.^135^

##### Tumor microenvironment

The status of p53 in tumor cells has a profound impact on the immune microenvironment. p53 regulates the release of cytokines and stimulates macrophage polarization toward the M1 phenotype to suppress tumorigenesis. Macrophages lacking p53 polarize toward M2 and enhance the proliferation of precancerous cells.^136^ p53 activation stimulates cellular anti-tumor responses,^137,138^ leading to interferon production, and there is a synergistic effect between cancer immunotherapy.^139^ Deletion or mutation of p53 in cancer affects the recruitment and activity of T cells, leading to immune evasion of cancer cells.^47,140^ Restoration of p53 expression enhanced the antitumor effect of anti-PD-1 monoclonal antibody on hepatocellular carcinoma cells and effectively induced reprogramming of the tumor microenvironment.^141^ *TP53* mutations are associated with increased expression of PD-L1, which may be a predictor of response to PD-L1 targeted checkpoint inhibitors.^142^ Mutant *TP53* contributes to the regulation of tumor cells into an immune microenvironment that is conducive to growth.^143–146^

##### Cancer stem cell self-renewal

The Rb/p53 signaling pathway regulates the proliferation and self-renewal process of neuroendocrine cells in the early stages of injury.^116^ Neuroendocrine cells return to a quiescent state after completing a limited proliferation.^147^ However, under conditions of Rb/p53 functional deficiency, neuroendocrine cells can acquire a sustained proliferative function and develop into small cell lung cancer.^148,149^ Activation of p53 in breast,^150^ prostate,^151^ epidermis,^152^ central nervous system^153^ and hematopoietic stem cells^154–156^ hinders stem cell self-renewal, but the exact mechanism remains to be confirmed.^48^ p53 has an essential role in regulating normal and malignant stem cell differentiation and self-renewal. Conversely, mutant p53 contributes to the maintenance of cancer stem cells.^157^ Mutant p53 enhances the proliferation of cancer stem cells by regulating WASP-interacting proteins.^158^ Mutant p53 also enhances the self-renewal of hematopoietic cells by upregulating FOXH1.^159^

The epithelial-mesenchymal transition is a form of reprogramming of cancer cells.^160^ Under pathological conditions, epithelial cells transform into mesenchymal cells, enhancing the invasive metastasis of cancer cells. p53 directly activates miR-200, miR-130 and miR-34 and inhibits the transcription factors Slug, Snail1 and Zeb1, which promote epithelial mesenchymal transformation.^161–164^ p53 deletion enhances self-renewal of lung cancer stem cells.^165^

##### Autophagy

Autophagy degrades intracellular macromolecules through the lysosomal pathway, thereby enabling intracellular energy supply and organelle renewal.^166,167^ p53 can directly activate damage-regulated autophagy regulators and induce autophagy.^168,169^ Autophagy is controlled by autophagy-related genes (Atg).^170^ Combined ChIP sequencing and RNA sequencing analysis revealed that p53 binds to many autophagy genes, including Atg2, Atg4, Atg7 and Atg10.^171^ This suggests that p53 induces autophagy in a transcription-dependent manner. Cathepsin D is a lysosomal aspartate protease and overexpression of cathepsin D activates autophagy.^172,173^ p53 binds to two DNA sites in the cathepsin D promoter region and regulates Cathepsin D expression.^174,175^ The upregulation of TGM2 expression by p53 enhances autophagy, thereby inhibiting oncogenic transformation and tumor formation in primary human mammary epithelial cells.^176^

p53 inhibits mTOR and enhances autophagy by activating AMP-responsive protein kinase.^177–179^ The p53 target genes Sestrin1 and Sestrin2 phosphorylate and activate AMPK and promote autophagy.^178^ p53 induces mitophagy by increasing the level of BNIP3.^180^ Interaction of beclin1 with Bcl-2 family anti-apoptotic proteins inhibits autophagy.^181,182^ p53 promotes the expression of BH3-only protein, which competitively binds to Bcl-2 family anti-apoptotic proteins, contributing to the restoration of beclin1 activity and promoting autophagy.^181,183^

p53 also functions as an inhibitor of autophagy. When p53 with deletion of nuclear localization sequence accumulates in the cytoplasm, it inhibits autophagy.^184,185^ HMGB1 and p53 regulate autophagy by mutually regulating their distribution in the nucleus and cytoplasm.^186,187^ In addition, autophagy regulates p53 activity. Atg7 inhibits p53 activation and p53 induced apoptosis.^188^

##### Other cellular processes

The signaling pathways involved in the regulation of p53 also include immune responses, non-coding RNAs and so on that exert tumor suppressive effects.^49,87,189–192^ In the process of tumor development, p53 suppresses tumor transformation,^22^ proliferation,^193^ metastasis^194^ and drug resistance^195^ through multifaceted regulation. p53 has an exceptionally flexible biological response that is altered by changes in cell type, cell differentiation status, stress conditions, and different signals in the environment.

#### Oncogenic effects of wild-type p53

**Fig. 3:**
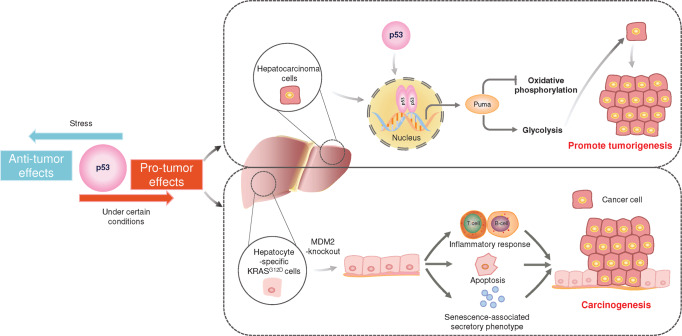
Pro-tumor effect of p53. In general, p53 is thought to have tumor suppressive effects, but in some cases, p53 promotes tumor growth. In hepatocellular carcinoma cells, p53 transcription activates the expression of Puma, which causes shift in mitochondrial energy metabolism from oxidative phosphorylation to glycolysis, thereby promoting tumorigenesis. In hepatocyte-specific KRAS^G12D^ cells knockout of MDM2 to activate p53. Accumulated p53 increased inflammatory responses, hepatocyte apoptosis, and senescence-associated secretory phenotypes that promote carcinogenesis

Studies have confirmed that p53 is essential for suppressing cancer in humans. However, a study in 2019 showed that p53 could promote tumor growth by enhancing the metabolism of hepatocellular carcinoma cells^117^ (Fig. 3). p53 transcription activates Puma expression, which further initiates apoptosis.^69^ But certain levels of Puma protein interfere with normal mitochondrial function, leading to a shift in mitochondrial energy metabolism from oxidative phosphorylation to glycolysis.^117^ Another group knocked out MDM2 in hepatocyte-specific KRAS G12D mutant mice and observed p53 accumulation in mouse hepatocytes.^196^ These p53-activated mice exhibited increased inflammatory responses, hepatocyte apoptosis, and senescence-associated secretory phenotype, leading to the facilitation of a carcinogenic microenvironment^196,197^ (Fig. 3). Hepatic progenitor cells from p53-accumulating mice were injected into experimental mice growing tumors. The development of hepatocellular carcinoma and other related phenotypes no longer occurred after knockdown of *TP53*, suggesting that p53-accumulated mice do promote the development of hepatocellular carcinoma.^196^ Cellular activities regulated by p53 are integrated into tumor suppressive functions, but p53-induced regulation of certain elements may also provide a survival advantage for tumors (Fig. 3).

### *TP53* mutations in cancer

**Fig. 4:**
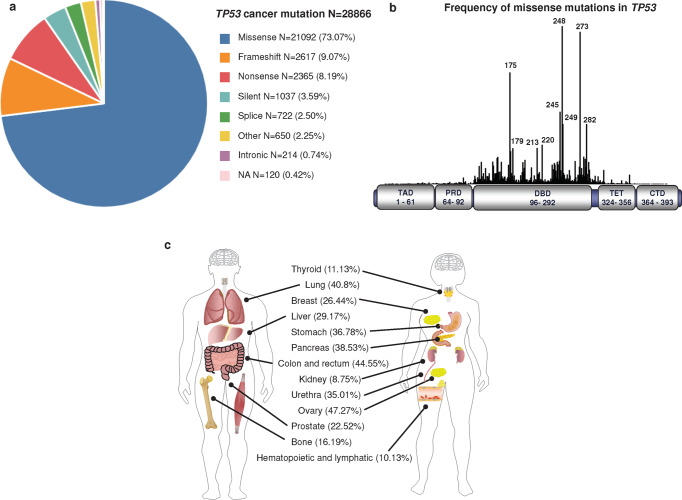
*TP53* mutations in cancer. **a** Frequency of somatic *TP53* mutations associated with different types of cancer. **b** Frequency of missense mutations in *TP53* (https://tp53.isb-cgc.org/). **c** Mutation frequency of *TP53* in different tissues and organs

The *TP53* gene is mutated in most tumor cells. Genome sequencing of different human cancer cells showed that 42% of cases carry *TP53* mutations.^198^ The major type of mutation in *TP53* is a missense mutation, a single amino acid substitution and the DNA binding domain (DBD) is the most common mutated region^199,200^ (Fig. 4a, b). p53 mutants are usually classified as structural mutants and DNA contact surface mutants. Structural mutants (R175H, R249S, G245S, Y220C) have reduced protein thermostability, resulting in proteins that do not fold properly at physiological temperatures and lose the ability to bind to DNA. Among them, R175H and C176Y affect the binding of protein to zinc ions. DNA contact surface mutants (R273H/C, R248W) are located in the DNA core binding region with mutations that prevent the binding of protein to DNA. R175, G245, R249, R282, R248 and R273 are the most common mutation sites and are therefore referred to as “hot spot” mutations of *TP53*^201–204^ (Fig. 4b). These mutants not only bind to wild-type p53 to produce dominant-negative (DN) effects but may also be converted to oncogenic proteins via gain-of-function (GOF).^203,205,206^ Thus, *TP53* differs from many “classical” oncogenes, which are usually characterized by nonsense or shift mutations that result in truncated protein inactivation.^207–210^

*TP53* mutations are prevalent in tumors, but different tissues and organs have different *TP53* mutation spectra^3^ (Fig. 4c). *TP53* mutations were commonly found in the ovary (47.27%), colon and rectum (44.55%), lung (40.8%), pancreas (38.53%), stomach (36.78%), urethra (35.01%), liver (29.17%), breast (26.44%), prostate (22.52%), bone (16.19%), thyroid (11.13%), hematopoietic and lymphatic (10.13%) and kidney (8.75%) (https://cancer.sanger.ac.uk/cosmic). *TP53* was mutated more frequently in esophageal carcinoma (93.77%), small cell lung cancer (79.06%), ovarian carcinoma (80.46%), colorectal carcinoma (74.45%) and gallbladder carcinoma (57.77%), and less frequently in thyroid carcinoma (3.13%), embryonal tumor (2.08%) and peripheral nervous system (1.25%). Different tumor subtypes of the same tissue and organ also have different *TP53* mutation spectra. For example, the frequency of *TP53* mutations in non-small cell lung cancer was 57.04%, which was lower than that in small cell lung cancer (79.06%) (https://www.cbioportal.org/). In addition, *TP53* mutation spectra differed by race. The frequency of *TP53* mutations in breast cancer is 42.9% in Asian and 30–35% in Caucasians.^211^

#### Mechanism of action of mutant p53

**Fig. 5:**
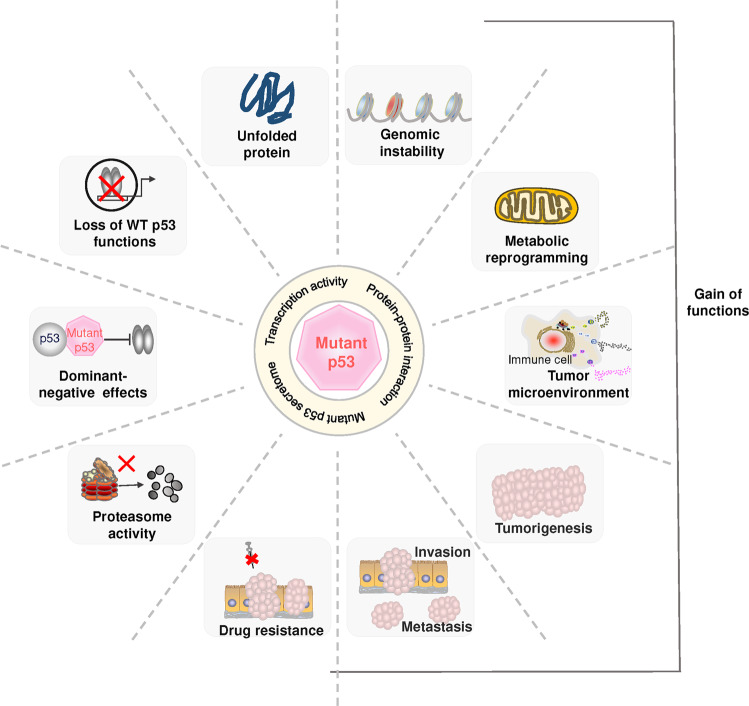
The role of mutant p53 in cancer. Mutant p53 can result in loss-of-function of wild-type p53, dominant-negative repression of wild-type p53 by mutant p53, and gain-of-function with oncogenic properties. Mutant p53 affects various cellular responses, such as genomic instability, metabolic reprogramming, and tumor microenvironment, and promotes cancer cell proliferation, invasion, metastasis and drug resistance. WT wild-type

*TP53* acts as a tumor suppressor gene and genome guardian, so cancer cell transformation is unlikely to occur in cells that maintain normal p53 function.^212,213^ *TP53* mutations provide a permissive environment for tumorigenesis.^214,215^ *TP53* mutations are a hallmark of an inherited cancer susceptibility syndrome known as Li-Fraumeni.^216,217^ The high frequency of *TP53* mutations found in tumor cells^19,218,219^ may be the result of selection pressures that favor tumor cells to escape surveillance and be spared from death.^220,221^ Many mutant p53 proteins are more stable than wild-type p53 proteins and can accumulate in cells. Some mutant p53 proteins may have completely different functions than wild-type proteins,^222^ and this effect may be due to altered target gene profiles, mutant p53 secretome or inappropriate protein-protein interactions^223^ (Fig. 5).

Loss of p53 transcriptional activity of mutant p53, and to some extent the DN effect are major drivers of the tumor phenotype (Fig. 5). The loss-of-function or DN effect of wild-type p53 enhanced the viability of tumor cells, and this effect was independent of missense mutations in *TP53*.^193^ The deletion of *TP53* in gastric cells combined with an oncogenic diet confers a selective advantage to cancer cells.^224^ Analysis of more than 10,000 patient samples from 32 different cancers in The Cancer Genome Atlas revealed that more than 91% of mutant p53 was accompanied by deletion of *TP53* alleles.^225^ Mutations in *TP53* are associated with enhanced chromosomal instability,^226,227^ and are accompanied by amplification of oncogenes and deletion of suppressor genes.^227,228^ Significant upregulation of proteins associated with cell cycle progression was also observed in p53 mutant cancers, which may be due to loss of control of cell cycle checkpoints by *TP53* deletion.^225,229,230^ Scott W. Lowe’s group used a mutation tracking system to reveal a pattern of *TP53* inactivation leading to genomic alterations.^231^ Although *TP53*-deficient cells gain the potential for cancer cell transformation, *TP53* deletion alone is not sufficient to cause cancer.^231^ *TP53*-deficient cells gain additional gene amplification in an ordered way that eventually spirals out of control and develops into cancer.^231–234^ Functional, DNA binding and transcriptional analyses against myeloid malignancy cell lines carrying *TP*53 missense mutations showed loss of p53 function, indicating that DN is the primary choice for *TP53* missense mutations in myeloid malignancies.^235^

The activity of the p53 mutant GOF has been reported to be associated with cellular physiopathology and poor clinical outcomes in cancer patients^236–246^ (Fig. 5). Mutant p53 binds directly to TBK1, prevents TBK1 from forming a ternary complex with STING and IRF3, and ultimately inhibits the activation of the cGAS-STING pathway.^223^ Mutant p53 helps tumor tissue evade the killing effect of the immune system by inhibiting the anti-tumor immune response.^223,247,248^ However, in contrast, the evidence on the effects of p53 mutant GOF is much weaker compared to DN, and it may act modestly or only in certain specific situations.

Mutated *TP53* not only lost its normal biological function, but also promoted cancer metastasis. Missense mutations in *TP53* were associated with lymph node metastasis in prostate cancer patients.^249^ *TP53* R175H and R273H mutations occurred in more metastatic tumors than in *TP53* knockout mice.^250,251^ In mouse models of pancreatic cancer that specifically express oncogenic KRAS and mutant *TP53*, more than twice as many metastatic lesions were observed as in *TP53* knockout mice.^252^ Lung adenocarcinoma mice carrying *TP53* and KRAS mutations are highly aggressive and metastasize to multiple sites of intrathoracic and extrathoracic in a pattern similar to that of human lung cancer.^253^ Similarly, *TP53* gene deletion induces an increase in systemic neutrophils, which drive systemic inflammation with breast cancer cell metastasis.^194^

Mutant p53 can lead to treatment resistance in cancer. Multidrug resistance protein 1 (MDR1, also known as P-glycoprotein), encoded by ATP-binding cassette subfamily B member 1, has been shown to be resistant to cytotoxicity and chemotherapy.^254^ In p53 mutant R248Q-expressing Hep3B cells, expression and activity of the multiple drug resistance gene, P-glycoprotein, are elevated mediating doxorubicin resistance.^255^ Acetylated mutant p53 interacts with p300 to promote transactivation of ephrin-B2 and enhances ATP-binding cassette subfamily G member 2 levels, thereby promoting chemoresistance.^256^ In R273H-expressing human squamous cell carcinoma cells, multidrug resistance to doxorubicin, methotrexate and apoptosis-inducing drugs was shown due to downregulation of procaspase-3 levels.^257^ Wild-type p53 inhibits LRPPRC expression via miR-34a, further reducing MDR1 expression. However, when *TP53* is mutated, chemotherapy-induced inactivation of this pathway and the accumulation of LRPPRC and MDR1 promote drug resistance.^195^ Mutated p53 binds to the miR-223 promoter and reduces its transcriptional activity, and the introduction of exogenous miR-223 makes tumor cells carrying mutated *TP53* sensitive to treatment.^258^

## The structures of p53

The comprehensive function of p53 is closely related to its modular structure of typical signaling proteins.^201^ Human p53 is a multidomain protein that consists of 393 amino acids.^259^ It contains an N-terminal transactivation domain (TAD, residues 1–61), a proline-rich domain (PRD, residues 64–92), a DNA-binding domain (DBD, residues 96–292) linked to a tetramerization domain (TET, residues 324–356), and a C-terminal regulatory domain (CTD, residues 364–393)^260,261^ (Fig. 4b). More than 40% of the regions within p53 are intrinsically disordered, including the TAD, CTD, and the linker between DBD and TET.^262^ These disordered regions allow p53 to interact as a modular protein with a wide range of partner proteins. These structured and unstructured domains individually have unique properties and contribute to the overall functional diversity and complexity of p53. Due to the intrinsic flexibility of the intact protein, the high-resolution structure of full-length p53 remains unclear.

### N-terminal region

**Fig. 6:**
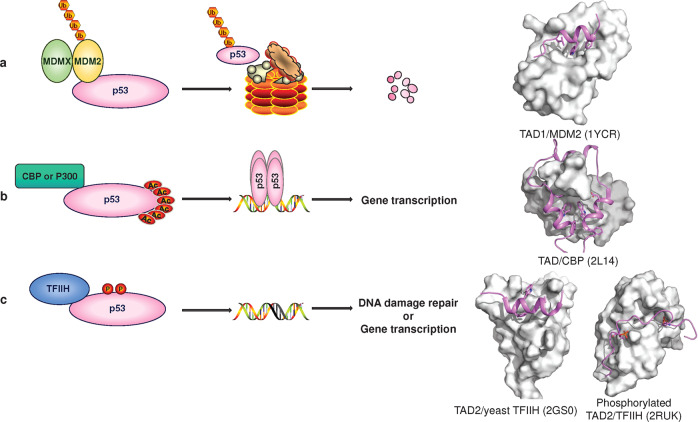
TAD binds to partner proteins and regulates transcription. **a** MDM2/MDMX is the major negative regulator of p53, and the C-terminus of MDM2 has E3 ubiquitin ligase activity (Ub) that promotes p53 degradation. **b** Under cellular stress conditions, the acetyl group (Ac) is added to the lysine residue of p53-CTD, and p53 binds to the target DNA sequence and interacts with the coactivator CBP or p300 to jointly promote gene transcription. **c** DNA damage and other stresses induce p53 phosphorylation (P) and binding to TFIIH, which stabilizes p53 and promotes DNA binding and transcription. p53-TAD shown as cartoon. Partner proteins are shown as surfaces

The N-terminal region of p53 contains a highly acidic, intrinsically disordered TAD and a proline-rich region.^263^ TADs, including TAD1 (residues 1–40) and TAD2 (residues 40–61),^264^ can bind to transcription machinery components and transcriptional coactivators to promote transcriptional initiation and interact with negative regulators to suppress transcriptional activation. TAD can adopt an amphipathic α-helical conformation upon binding of partner proteins,^259^ which seems to be an essential binding mode, as seen in the structures of TAD bound to the negative regulator MDM2^265^/MDMX^266^ (Fig. 6a), transcription coactivator CBP^267,268^/p300^269,270^ (Fig. 6b), replication protein A,^271^ high-mobility group B1, HMGB1,^272^ Tfb1 subunits of yeast TFIIH^273^ and metastasis-associated protein S100A4.^274^ Notably, phosphorylated TAD2 exhibits an acidic string-like conformation when bound to subunit p62 of human TFIIH^275^ (Fig. 6c). Phosphorylation of TAD has been shown to serve as a switch to rapidly turn p53 function on and off but also as a mechanism for a graded p53 response.^276,277^ Overall, the conformational plasticity of the TAD, together with posttranslational modifications, makes p53 a highly efficient transcription factor.

The proline-rich region linking the TAD to DBD is required for p53 to induce cell cycle arrest and apoptosis.^278,279^ This region plays a role in signal transduction by binding to Src homology 3 domains.^280^ The PRD is also involved in interactions with focal adhesion kinase (FAK),^281^ peptidase D,^282^ ASPP family members,^283^ and the corepressor protein mSin3a.^284^ The PRD contains five PXXP motifs, some of which may adopt a polyproline helix-like structure.^259^ The exact structure and interaction mechanism of PRD are largely unknown.

### DNA-binding domain

**Fig. 7:**
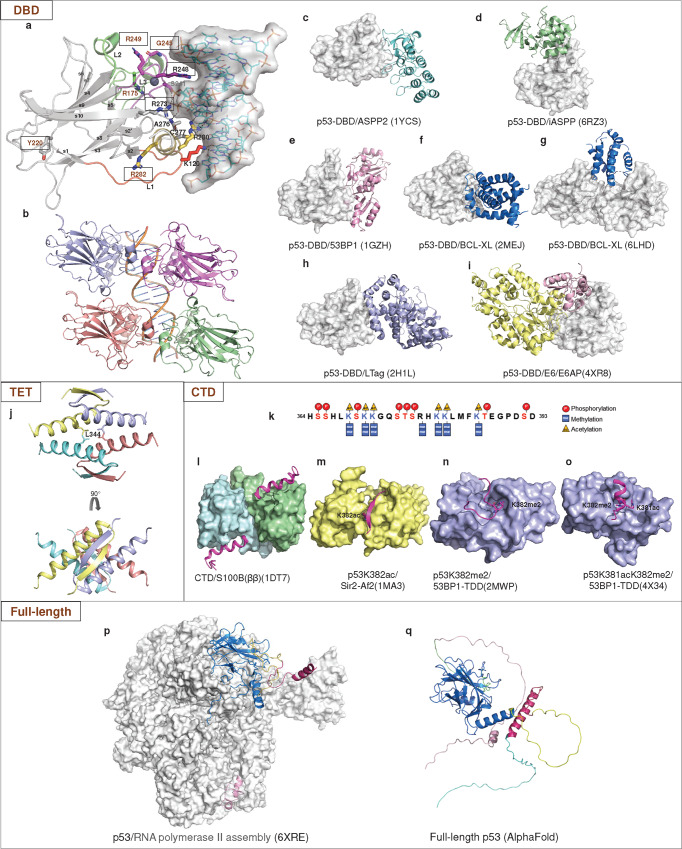
Structures of DBD, TET, CTD and full-length p53. **a** The structure of DBD in complex with a sequence-specific DNA (PDB: 1TSR). p53-DBD is shown as a cartoon, and the secondary structures are labeled. The interfacial residues are shown as sticks. DNA is shown as sticks and surfaces. **b** DNA recognition by the p53 tetramer (PDB: 3KMD). **c** ASPP2 (colored aquamarine). **d** iASPP (colored pale green). **e** 53BP1 (colored pink). **f** NMR structural model of the p53-DBD/BCL-xL complex. **g** Crystal structure of the p53-DBD/BCL-xL complex. BCL-xL is colored marine. **h** LTag (colored light blue). **i** E6/E6AP. E6 is colored light pink. E6AP is colored pale yellow. All p53-DBD molecules are shown as the surface. **j** Assembly of the p53 tetramerization domain (PDB: 1C26). **k** CTD sequence. The posttranscriptional modifications are shown as indicated. **l** A CTD peptide (colored light magenta) became a helical conformation when bound to Ca^2+^-loaded S100B(ββ) (colored pale cyan and pale green). **m** A CTD peptide dimethylated at K382 (p53K382me2) binds the tandem Tudor domain (TTD) of 53BP1 (colored yellow) in a U-shape conformation. **n** A p53 peptide acetylated at K381 and dimethylated at K382 (p53K381acK382me2) forms a helical conformation when interacting with 53BP1-TTD. **o** A p53K382ac peptide forms β sheet-like contacts with deacetylase Sir2-Af2 (colored light blue). **p** Structure of the p53/RNA polymerase II assembly. RNA polymerase II assembly is shown as surface. p53 is shown as cartoon. **q** Full-length p53 structure predicted by AlphaFold2. TAD colored pink, PRD colored green, DBD colored marine, Loop colored yellow, TET colored warm pink, and CTD colored cyan

The structured DBD adopts a basic scaffold of an immunoglobulin-like β-sandwich, a loop-sheet-helix motif (loops L1, S2, and S2’, parts of the extended S10 and H2), and two large loops (L2 and L3) that are stabilized by a tetrahedrally coordinated zinc ion^285^ (Fig. 7a). The DBD is responsible for binding sequence-specific target DNA, which is central to the biological function of p53 as a transcription factor. In addition, the DBD is also capable of interactions with diverse proteins.

#### DNA recognition

In response to a wide variety of stress signals, p53 regulates the transcription of many different genes involved in various pathways. p53 also acts as a pioneer transcription factor, binding to specific DNA sequences in the nucleosome to promote transcriptional activation in chromatin.^286–288^ Most p53 target genes contain a consensus response element (RE) composed of two decametric half-sites of RRRCWWGYYY (R = A, G; W = A, T; Y = C, T) separated by 0–13 base pairs.^289–292^ Some REs are cluster sites that have more than two half-sites. p53 also binds a set of noncanonical DNAs, such as cruciforms, left-handed DNA (Z-DNA), quadruplex and triplex DNA.^293,294^

Various structural studies have demonstrated the molecular mechanism of DNA recognition by p53 (Fig. 7a). Briefly, the residue Arg248 from the large loop L3 interacts with the minor groove.^285^ The loop-sheet-helix motif docks into the major groove and makes hydrogen bond contacts with bases via key residues Lys120, Cys277 and Arg280.^285^ Residues Ala276, Ser241 and Arg273 contribute to DNA backbone contacts. p53 binds to a full consensus DNA response element as a tetramer through a highly cooperative self-assembling mechanism (Fig. 7b). Two DBDs bind each decameric half-site as a symmetrical dimer (A-B), and two such dimers (A-B and C-D) constitute a tetramer on a full DNA response element via protein-protein and base stacking interactions.^11,70,295–299^

Upon DNA binding, the DBD and the DNA helix undergo structural changes. The binding of p53-DBD to the BAX response element causes DNA deformation, which is partially disordered around the spacer region, leading to unwinding and compression of the region to allow protein-protein interactions.^70^ In some p53/DNA structures, the central A-T doublet of each half-site shows noncanonical Hoogsteen base-pairing geometry instead of standard Watson-Crick base pairs.^299,300^ Some p53/DNA structures reveal that loop L1 of two p53 subunits adopts a conformational switch from an extended conformation where Lys120 interacts directly with DNA to a recessed conformation where there is no direct DNA contact.^301^ The conformational switch is related to the DNA-binding specificity of p53. Acetylation of loop L1 residue Lys120 expands the conformational space of loop L1 in the DNA-bound state and promotes sequence-dependent DNA-binding modes for p53.^302^

#### DBD-mediated protein-protein interactions

In addition to DNA recognition, the DBD mediates protein-protein interactions with multiple proteins. Interactions with these proteins influence various activities of p53. An increasing number of structures of the DBD/protein complex have been determined, providing fresh perspectives on the mechanisms of p53-DBD binding and functional diversity.

By using a yeast two-hybrid system, 53BP1 and 53BP2 (namely, the C-terminal fragment of ASPP2) were initially identified to bind to p53.^303^ They interact with p53-DBD through the L3 loop and L2 loop^304–306^ (Fig. 7c, e). 53BP1 plays multiple roles in DNA damage and repair and has been reported to enhance p53-mediated transcriptional activation of ASPP2. The inhibitory member iASPP belongs to the apoptosis-stimulating p53 protein (ASPP) family, which has opposite functions in regulating the apoptotic function of p53.^307^ Unlike ASPP2, iASPP preferentially binds p53-PRD^283^ and interacts with the DBD through the L1 loop, helix H2 and N-terminal loop^308^ (Fig. 7d). The L1 loop of p53 moves away from other DNA-binding modules upon binding iASPP, which disables Lys120 from making contact with a specific base.^308^ The different binding interfaces with the ASPP family provide insight into the opposing regulatory mechanism of p53.

Cytoplasmic p53 has been reported to regulate the mitochondrial apoptosis pathway by inhibiting antiapoptotic BCL-2 and BCL-xL. In a structural model of BCL-xL/p53 characterized by nuclear magnetic resonance (NMR) spectroscopy and determined using the HADDOCK docking method based on 1:1 stoichiometry, the BCL-xL binding surface of p53-DBD largely overlaps with the DNA-binding surface and encompasses helix H1 and the Zn^2+^-coordination site^309^ (Fig. 7f). However, the recently determined crystal structure of the p53/BCL-xL complex indicates that p53 binds BCL-xL with a 2:1 stoichiometry^71^ (Fig. 7g). Two p53-DBD molecules dimerize through the N-terminal loop and β9-β10 loop of one p53-DBD and the Zn^2+^-coordination site of the other p53-DBD, involving residues Tyr107 and His178, respectively. The resulting p53-DBD dimer forms a groove and interacts with one BCL-xL to form a ternary complex. The DNA-contacting residues Arg248 and Arg273 form direct hydrogen bonds with BCL-xL. The binding mode is distinct from other p53-DBD binding proteins.

The p53-DBD is also targeted by viral oncoproteins, such as the large T antigen (LTag) of simian virus 40 (SV40)^310^ (Fig. 7h) and human papillomavirus (HPV) oncoprotein E6^311,312^ (Fig. 7i). LTag promotes viral replication and cellular transformation. It occupies the entire DNA-binding surface of p53-DBD and interacts with a region of helix H1 that is involved in p53-DBD dimerization. The p53-binding site of HPV E6 substantially overlaps with the binding surface of iASPP, which has been reported to inhibit HPV E6-induced degradation of p53.^308^

Some of these proteins bind p53 at a surface overlapping with the DNA-binding site, such as 53BP1, ASPP2, Bcl-xL and LTag. Some partner proteins bind at a surface distal from the DNA-binding site, such as iASPP and HPV E6. Notably, the p53 hotspot mutant alleles at residues Arg248 and Arg273 not only make direct contact with DNA but are also involved in direct contact with ASPP, BCL-xL and LTag. Another hotspot residue, Arg282, is involved in iASPP binding. Thus, these hotspot mutants may have multiple hits on p53 activities.

### Tetramerization domain

Full-length p53 reversibly forms tetramers through the TET. The structural analysis demonstrated that monomeric TET is a V-shaped structure composed of a β-strand and an α-helix linked by hinge residue Gly344^313–315^ (Fig. 7j). The primary dimer relies on eight backbone hydrogen bonds of the β-sheet and is also stabilized by hydrophobic interactions (Phe328, Leu330, Ile332, Phe338 and Phe341) as well as a salt bridge between Arg337 and Asp352.^35^ The dimer-dimer interactions are primarily mediated by hydrophobic contacts (Met340, Phe341, Leu344, Ala347, Leu348 and Leu350).^35^ R337C/H/P and L344P affect the formation and transcriptional activity of p53 tetramers and are involved in Li-Fraumeni Syndrome and Li-Fraumeni-Like Syndrome.^316^ The G334V mutant contributed to a beta-dominated structural transition leading to amyloid formation at physiological temperature.^317^ A highly conserved leucine-rich nuclear export signal (NES) within the TET is necessary for the subcellular localization of p53.^318^ Some proteins bind directly to TET, but no complex structure has yet been demonstrated.^319^

### The C-terminal regulatory domain

Similar to the NTD, the CTD is also an intrinsically disordered region. This characteristic endows it with multiple regulatory functions in almost every aspect of p53, including DNA binding, cofactor recruitment, cellular localization, and protein stabilization.^320^ The CTD possesses many positively charged residues, which are highly conserved in mammals. Extensive posttranslational modifications of the CTD, including acetylation, methylation and phosphorylation, play an essential and interdependent role in its function and stability (Fig. 7k). The CTD adopts different conformations depending on the binding partners and posttranslational modifications^201,321^ (Fig. 7l–o).

### Full-length p53

The highly intrinsic unfolded regions within p53 make it difficultt to determine the high-resolution structure of full-length p53. A 4.6 Å resolution structure of RNA polymerase II (Pol II) with full-length p53,^322^ has shown that the DBD targets the upstream DNA-binding site within Pol II, and the following TET is exposed on top of the DBD. The TAD distal to the DBD forms helices and binds Pol II’s jaw that contacts downstream DNA (Fig. 7p). This association introduces a conformational change in Pol II, providing insight into the p53-mediated regulation of gene expression.

How an intact p53 tetramer interacts with DNA and proteins is still largely unclear. A combination of NMR, electron microscopy, small-angle X-ray scattering, and FRET techniques indicates that the free p53 tetramer in solution forms an open cross-shaped structure with a pair of loosely coupled DBD.^323,324^ The oligomers close to form a compact complex upon binding a specific DNA response element. Chemical cross-linking and mass spectrometry methods are also employed to study the structural dynamics of full-length p53.^325^ A recent study has reported cryo-electron microscopy of p53-DBD and full-length p53 complexed with a nucleosome, in which the DBD binds as a tetramer to the DNA and peels the DNA from the histone surface.^288^ The N-terminal and C-terminal regions were not observed in the cryo-EM maps, but biochemical analysis suggests that the C-terminus of p53 may contain an additional DNA binding domain.^288^

The artificial intelligence system AlphaFold has accomplished the great challenge of protein amino acid sequence to structure, bringing great advances to the field of biology, especially structural biology.^326–328^ In 2021, AlphaFold2 released its predicted structure of the full-length p53 protein (https://alphafold.com/) (Fig. 7q).^329^ The predicted full-length p53 structure is shown as a monomer with the DBD and TET regions resembling the known structures of individual structural domains. Nevertheless, it does not provide more information on how the disordered regions fold and how p53 assembles into a tetramer. In addition, the most important feature of p53 is missense mutations leading to its altered or lost function. AlphaFold2 is currently unable to correctly predict the structural impact of missense mutations.^330^ Furthermore, the p53 protein functions by binding to various ligands such as DNA, small molecules, metal ions or other proteins. We still lack structural information about this interaction to aid in drug development against p53 and recognition of protein complexes.^331–333^ Following technological revolution, we expect that artificial intelligence will provide more valuable information in the future.

Regardless, structural studies on full-length p53 in complex with different DNA targets, full-length partner proteins, and even higher-order complexes are needed. This structural biology information will not only help to understand the role of p53 in cellular life activities, but these structures may also help to design drugs that target p53.

## Structure-based p53 targeting

Structural and biochemical analyses have provided insight into the function of p53. However, many important questions regarding the structure‒function and specific regulation of p53 remain to be determined. In recent years, with the development of NMR, protein crystallography and cryo-electron microscopy techniques, much structural information has been obtained about the binding of p53 to ligands. From this critical information, structure-based design strategies can be applied to optimize the structure and thus improve activity and selectivity.

### MDM2/MDMX-p53, a central hub for p53 activation

**Fig. 8:**
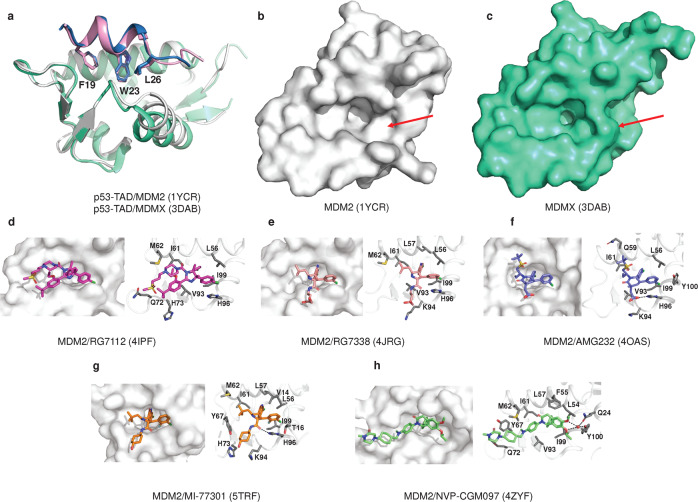
Structure of MDM2/X with small molecules. **a** Overlay of the crystal structures of MDM2/p53-TAD (white, sky blue), MDMX/p53-TAD (greencyan, pink) and the three residues of p53-TAD (F19, W23, L26) are shown as sticks. **b** MDM2 is shown as a surface. **c** MDMX is shown as surface. **d** MDM2/RG7112 (PDB: 4IPF). **e** MDM2/RG7388 (PDB: 4JRG). **f** MDM2/AMG 232 (PDB: 4OAS). **g** MDM2/MI-77301 (PDB: 5TRF). **h** MDM2/NVP-CGM097 (PDB: 4ZYF). Water molecules are red spheres, and hydrogen bonds are black lines. The interacting amino acid residues are shown as sticks (colored gray)

MDM2/MDMX is the major negative regulator of p53.^334^ In the MDM2-p53 negative feedback regulatory loop, wild-type p53 activates the transcription of MDM2, while the N-terminal of MDM2 binds to p53-TAD and represses the transcriptional activity of p53.^265^ In addition, the C-terminus of MDM2 has the enzymatic activity of E3 ubiquitin ligase, which promotes the nuclear export and degradation of p53.^335,336^ MDMX (or MDM4), a homolog of MDM2, is structurally similar to MDM2, but it lacks ubiquitin ligase activity and can enhance the ubiquitination activity of MDM2 by forming a dimer with MDM2.^337,338^ Overexpression or activation of MDM2/MDM4 is present in many human tumors, leading to p53 inactivation.^334,339,340^ Many antitumor drugs targeting MDM2/MDMX-p53 interactions have been developed in recent years (Fig. 8, Table 1).

**Table 1:** MDM2/X inhibitor in clinical trails

Inhibitors	Chemical class (first report time)	Chemical structure	Affinity	Population	Identifier /status/phase
RG7112	Imidazoline (2013)		Kd = 2.9 nM	Hematologic neoplasms, advanced solid tumors, PV or ET	NCT00623870 Completed I NCT00559533 Completed I NCT01677780 Completed I NCT01164033 Completed I NCT01605526 Completed I NCT01143740 Completed I NCT01635296 Completed I
RG7388	Imidazoline (2013)		Kd = 0.15 nM	AML, ALL, ET or PV, FL, MM, DLBCL, solid tumors	NCT03287245 Terminated II NCT02545283 Terminated III NCT03362723 Completed I NCT02828930 Completed I NCT02633059 Active, not recruiting I/II NCT03850535 Terminated I/II NCT02407080 Completed I NCT03135262 Terminated I/II NCT02624986 Terminated I/II NCT04029688 Recruiting I/II NCT02670044 Completed I NCT03566485 Terminated I/II NCT04589845 Recruiting II NCT03555149 Active, not recruiting I/II NCT03158389 Recruiting I/II
AMG232	Piperidone (2014)		Kd = 0.045 nM	AML, MM, solid tumors	NCT04640532 Recruiting I/II NCT04835584 Recruiting I/II NCT04502394 Recruiting I/II NCT05027867 Recruiting II NCT04485260 Recruiting I/II NCT03787602 Recruiting I/II NCT04878003 Recruiting II NCT04669067 Recruiting I/II NCT04113616 Recruiting I/II NCT03669965 Active, not recruiting I NCT03217266 Active, not recruiting I NCT03662126 Recruiting II/III NCT04190550 Recruiting I NCT03031730 Recruiting I NCT03107780 Suspended I NCT03041688 Suspended I
SAR405838	Spirooxindole (2014)		Ki = 0.88 nM	Neoplasm malignant	NCT01636479 Completed I NCT01985191 Completed I
NVP-CGM097	Dihydroisoquinoline (2015)		Ki = 1.3 nM	Advanced solid tumors	NCT01760525 Completed I
HDM201	Imidazopyrrolidinone (2016)		In the picomole range	AML, MDS, sarcoma, solid tumors	NCT05180695 Recruiting I/II NCT04496999 Recruiting I NCT03714958 Recruiting I NCT02343172 Completed I NCT02143635 Completed I NCT03940352 Recruiting I NCT03760445 Withdrawn I/II NCT02601378 Terminated I NCT05447663 Not yet recruiting I/II NCT05155709 Recruiting I/II NCT04097821 Suspended I/II NCT04116541 Recruiting I NCT02890069 Completed I
MK-8242	Purine carboxylic acid-derived inhibitor (2016)		IC_50_ 0.07 μM	AML, solid tumors	NCT01451437 Terminated I NCT01463696 Terminated I
DS-3032	Dispiropyrrolidine (2017)		IC_50_ 17.7 ± 5.1 nM	AML, MDS, MM, lymphoma, advanced solid tumor	NCT02579824 Terminated I NCT03634228 Completed I/II NCT03671564 Completed I NCT03552029 Terminated I NCT02319369 Terminated I NCT01877382 Completed I
APG-115	Spirooxindole (2017)		Ki 60 ± 22 nM	AML, CMML, MDS, T-PLL, lymphoma, sarcoma, advanced solid tumor	NCT02935907 Completed I NCT04496349 Recruiting II NCT04785196 Recruiting I/II NCT04358393 Recruiting I/II NCT03611868 Recruiting I/II NCT03781986 Recruiting I/II NCT04275518 Recruiting I
BI-907828	Unknow (2020)	–	IC_50_ 58.52 pM	Liposarcoma, solid tumors	NCT05372367 Recruiting I NCT05376800 Recruiting I NCT05512377 Not yet recruiting II NCT05218499 Recruiting II/III NCT03964233 Recruiting I NCT03449381 Recruiting I
ALRN-6924	Stapled peptide (2018)	–	MDMX Kd 57 nM MDM2 Kd 10.9 nM	AML, MDS, lymphoma, solid tumor	NCT02909972 Completed I NCT03725436 Recruiting I NCT03654716 Recruiting I NCT02264613 Completed I/II NCT04022876 Active, not recruiting I

*ALL* acute lymphoblastic leukemia, *AML* acute myeloid leukemia, *CMML* chronic myelomonocytic leukemia, *DLBCL* diffuse large B-cell lymphoma, *ET* essential thrombocythemia, *FL* follicular lymphoma, *MDS* myelodysplastic syndrome, *MM* multiple myeloma, *PV* polycythemia vera, *T-PLL* T-prolymphocytic leukemia

#### MDM2-p53 inhibitor

The cocrystal structure of p53-TAD with MDM2 shows that Phe19, Trp23 and Leu26 on the α-helix of p53 penetrate deeply into the hydrophobic cleft of MDM2 (Fig. 8a, b), with Leu22 providing additional van der Waals forces.^265^ These structural features of the p53/MDM2 complex provide a basis for finding inhibitors that block the interaction between these two proteins.

##### RG7112

Based on the structural features of p53/MDM2, researchers synthesized imidazole-like MDM2 antagonists, among which RG7112 is the first small-molecule inhibitor of MDM2 to enter the clinic. The crystal structure showed that the 4-chlorophenyl ring occupied the Trp23 and Leu26 pockets, while the ethoxy group was prominently located in the Phe19 pocket^341^ (Fig. 8d). RG7112 had a Kd = 2.9 nM. It effectively blocks p53-MDM2 binding and promotes cancer cell cycle arrest and apoptosis.^342^ However, the ability of RG7112 to induce apoptosis in cancer cells varies widely. The best responses were observed in MDM2 gene-amplified osteosarcoma cell lines and xenografts.^341,343^

##### RG7388

The MDM2 inhibitor RG7388, which was subsequently designed and synthesized, is a class of pyrrolidine derivatives.^344^ RG7388, Kd = 0.15 nM, induces dose-dependent apoptosis in wild-type p53 cancer cells.^342^ Structurally, the 4-chlorophenyl ring, 3-chlorophenyl group and neopentyl group mimic p53 occupying the Phe19, Trp23 and Leu26 pockets. In addition, the 3-chlorophenyl group forms a π-π stacking interaction with the His96 residue on MDM2, and the pyrrolidine Cα carbonyl group forms another hydrogen bond with His96^344^ (Fig. 8e). RG7388 activates p53 to inhibit hematological tumors^345^ and solid tumors,^346,347^ but long-term administration may lead to p53 mutations and drug resistance.^348,349^

##### AM232

AMG32, Kd = 0.045 nM, is a piperidone analog that potently inhibits the MDM2-p53 interaction.^342^ Structural analysis revealed that the “cleft” of Gly58 may provide additional assistance for the small molecule to bind MDM2.^350^ The crystal structure shows that the isopropyl group, C6 aryl group and C5 aryl group occupy the three major binding pockets of p53, namely, Phe19, Trp23 and Leu26, respectively. Meanwhile, the imidazole group of His96 formed π-π stacking interactions with the C5 aryl group and formed hydrogen bonds with carboxylate.^342^ In addition, the isopropyl group extends into the “cleft” of Gly58, forming CH·O-type interactions with Gly58 and enhancing contacts with nearby hydrophobic residues^350^ (Fig. 8f). In tumor cells with high MDM2 expression, AMG232 enhances p53 activity and inhibits tumor growth.^351^ AMG232, in combination with immune checkpoint drugs, also enhances T-cell-mediated tumor killing.^352^ However, high doses of AMG232 may trigger gastrointestinal side effects, neutropenia and leukopenia, which requires further study.^353^

##### SAR405838

SAR405838 (MI77301), Ki = 0.88 nM, is an optimized spiro-oxide compound that blocks the MDM2-p53 interaction and prevents p53 degradation.^354^ SAR405838 mimics the three key amino acid residues of p53 and forms hydrogen bonds with the His96 residue of MDM2 at different chemical groups, generating π-π stacking, as seen in other MDM2 inhibitors. The structural differences are that SAR405838 mediates the refolding of the MDM2 N-terminus (residues 10–18) and interacts extensively with Val14 and Thr16. Moreover, the hydroxy-cyclohexyl group forms a hydrogen bond with Lys94^354^ (Fig. 8g). These structural features allow SAR405838 to achieve a tight binding and high specificity for MDM2. SAR405838 activates the p53 pathway, increases the expression of PUMA and P21, and induces complete tumor regression in SJSA-1 osteosarcoma xenograft mice.^354^ The safety of SAR405838 alone^355^ or in combination with MEK inhibitors^356^ has been established, but the drug activity is limited, and *TP53* mutations may occur with long-term administration.^357^

##### NVP-CGM097

NVP-CGM097, Ki = 1.3 nM, is a novel dihydroisoquinoline-like MDM2 inhibitor obtained by virtual screening and structural optimization.^358^ The cocrystal structure revealed that in addition to the three key amino acids that mimic the interaction between p53 and MDM2, the isopropyl ether group also forms water-mediated hydrogen bonds with Tyr100, Gln24 and Phe55. NVP-CGM097 induced a conformational change in Phe55; thus, Phe55 formed a π-π stacking interaction with the dihydroisoquinolone core (Fig. 8h). NVP-CGM097 exhibits high selectivity for wild-type p53 and shows potent antiproliferative ability in colorectal cancer and osteosarcoma cells with wild-type p53.^358^ NVP-CGM097, combined with MEK inhibitors, activates the MAPK signaling pathway and attenuates acute myelogenous leukemia (AML) cell load.^359^ When combined with BET or Cdk4/6 inhibitors, NVP-CGM097 induces cell death in neuroblastoma or ER-positive breast cancer cells.^360,361^

#### MDM2/MDMX-p53 inhibitor

The main features of the MDM2-p53 interaction are preserved in the MDMX-p53 structure, but the central hydrophobic cleft of p53 peptide binding in MDMX is smaller and differently shaped than that of MDM2 (Fig. 8a–c). Therefore, many small molecule drugs targeting MDM2 do not bind MDMX well.

##### ALRN-6924

Currently, there are no inhibitors that act on MDMX alone, but one stapled peptide, ALRN-6924, binds both MDM2 (Kd = 10.9 nM) and MDMX (Kd = 57 nM).^362^ In AML cells, ALRN-6924 induces cell cycle arrest and apoptosis and significantly prolongs the survival of AML model mice.^362^ ALRN-6924 is well tolerated in phase I clinical trials in patients with solid tumors and lymphomas carrying wild-type p53.^363^ However, molecular simulations reveal that ATSP-7041, an analog of ALRN-6924, may bind to the p53 coactivator p300 and isolate free p300, thus reducing the transcriptional activity of p53.^364^ This mechanism needs further investigation.

#### Other MDM2 inhibitors

Other MDM2 inhibitors currently in clinical trials include HDM201,^365,366^ MK-8242,^367–369^ BI-907828,^370^ APG-115,^371–373^ and milademetan (DS-3032b)^374^ (Table 1). Although the specific interaction sites are unknown, they all induce cell death in a variety of tumor cells, particularly in patients with MDM2 amplification and intact p53 expression. APG-115 was the first MDM2 inhibitor to enter the clinic in China and was granted Fast Track Designation (FTD) by the U.S. Food and Drug Administration. APG-115 interrupts the p53-MDM2 interaction, increases the abundance of MDM2 in T cells, and plays a critical biological role in maintaining T-cell stability, survival, and antitumor immunity.^371^

In addition, researchers designed Proteolysis-targeting chimera (PROTAC) degraders based on MDM2 inhibitors.^375^ WB156, consisting of a nutlin derivative linked to the CRBN ligand lenalidomide, effectively depleted MDM2 and activated wild-type p53, thereby inducing apoptosis.^376,377^ The rapid degradation of MDM2 by MD-224 resulted in complete tumor regression in leukemia cells carrying wild-type p53.^378^

Overall, the design and development of drugs targeting MDM2/MDMX-p53 is a hot spot and priority in the field of oncology drug research worldwide. However, the specificity of MDM2/MDMX-p53 protein interactions poses a great difficulty in the development of small molecule inhibitors. Although some MDM2 inhibitors have entered clinical trials, there are currently no marketed drugs, and it is hoped that structure-based guidance will lead to new breakthroughs for MDM2-p53 inhibitors.

### Tackling the p53 mutation

Under normal conditions, wild-type p53 suppresses tumor development through transcriptional regulation and protein-protein interactions. However, in many situations, missense mutant p53 is expressed at high levels in tumor cells, partly due to the inability of mutant p53 to induce gene expression of MDM2,^379^ which supports the reactivation of mutant p53 as a therapeutic option.^5^ Most *TP53* mutations are missense mutations located in the DBD.^3,4,203,205,380^ p53 mutants mainly affect the thermostability of p53 protein, structural stability (structural mutants such as 175, 220, 245 and 249) or p53-DNA contact (DNA contact mutants such as 248 and 273). Therefore, a number of small molecule compounds, peptide or antibody drugs targeting p53 mutants have been developed to recover the native conformation or normal function of the p53 protein.^381–385^

#### Broad-spectrum mutant p53 rescue compounds

Employing library screens, structure-based design and other methods, drugs or compounds were discovered to have effects on the thermostability, specific DNA binding capacity or transcriptional activity of p53 mutant proteins. Some of them were initially identified as p53 mutant activators, but as research progressed, it was discovered that they could also exert antitumor activity independent of p53 status (Tables 2–3).

**Table 2:** Drugs targeting mutant p53

Compounds	Discovery method (Report time)	Chemical structure	p53 targets	Rescue potency	Method: detailed interactions	Identifier/phase/status	Refs
**Broad-spectrum mutant p53 rescue compounds**							
CP-31398	Screening a chemical library (1999)		Null, WT and mutant p53	Thermostabilized, transcriptional activity	NMR: Does not bind p53	–	^454–456^
PRIMA	Screening a chemical library (2002)		Null, WT and mutant p53	Restore conformation and transcriptional activity	X-ray: Binds to cysteine	–	^386^
APR-246	Methylated form of PRIMA-1		Null, WT and mutant p53	Restore conformation and transcriptional activity	X-ray: Binds to cysteine	NCT03931291 Completed II NCT04214860 Completed I NCT04383938 Completed I/II NCT02999893 Terminated I/II NCT04419389 Suspended I/II NCT03588078 Unknown I/II NCT03745716 Completed III NCT03268382 Completed II NCT03391050 Terminated I/II NCT03072043 Completed I/II NCT02098343 Completed Ib/II NCT00900614 Completed I NCT04990778 Withdrawn II	^425^
STIMA-1	Cell-based screening (2008)		Null, WT, and mutant p53	Transcriptional activity	Docking: L1/S3 pocket, C124	–	^458^
MIRA-1	Cell-based screening (2005)		WT and mutant p53	Restore conformation and transcriptional activity	Docking L1/S3 pocket, C124	–	^459–461^
Stictic Acid	Screening a chemical library (2013)		Mutant p53	Thermostabilized, transcriptional activity	Docking: L1/S3 pocket, C124	–	^424^
ATO	Cell-based screening (2021)	As_2_O_3_	Structural mutants	Thermostabilized, transcriptional activity	X-ray: C124, C135, C141, M133	NCT04489706 Recruiting – NCT04695223 Recruiting II NCT03381781 Unknown II NCT03855371 Recruiting I NCT04869475 Recruiting II NCT03377725 Unknown III NCT01428128 Completed II	^431^
PAT	Cell-based screening (2022)	C_8_H_4_K_2_O_12_Sb_2_	Temperature-sensitive mutants	Thermostabilized, transcriptional activity	X-ray: C124, C135, C141, M133	NCT04906031 Recruiting II	^432^
ZMC1	Silico screen (2012)		Mutant p53	Restore conformation, Zinc ion delivery and transcriptional activity	-: Delivered zinc bound to C238, C242 and C176	–	^441^
COTI-2	Structure-based design (2016)		WT and mutant p53	Restore conformation and transcriptional activity	–	NCT02433626 Unknown I	^186^
UCI-LC0023	Virtual screen (2022)		Mutant p53	Restore conformation and transcriptional activity	Docking: L1/S3 pocket	–	^462^
**Allele-specific mutant p53 rescue compounds**							
PhiKan083	Structure-based in silico screening (2008)		Y220C/S Kd = 140 ± 73 μM	Thermostabilized, structural stability	X-ray: The ethyl group bound to sulfhydryl group of C220, N-methyl methanamine forms a hydrogen bond with D228, hydrophobic interaction: F109, V147, L145 L257.	–	^467,474^
PhiKan5196	Screening halogen-enriched fragment library (2012)		Y220C Kd = 9.7 μM	Structural stability	X-ray: An iodine atom forms halogen bond with L145, the phenol group forms water molecule-mediated hydrogen bonds with V47 and D228, benzamine moiety forms a hydrogen bond with C220, hydrophobic interactions: P153, P222.	–	^468^
PK7088 (PK7242)	Cell-based techniques (2013)		Y220C Kd, ~140 μM	Structural stability	X-ray: The flip of the C220 side chain increases the depth of the cavity, and the pyrazole ring penetrates deep into the pocket, while forming water molecule-mediated hydrogen bonds with L145, D228, hydrogen bonds with T230.	–	^469^
Compound9	Fragment screening or structure-based design (2015)		Y220C Kd = 21 μM	Structural stability	X-ray: The pyrrole moiety binds to the cavity of the C220 side chain flip. Iodine forms a halogen bond with L145, while forms hydrogen bonds with T150 and structural water molecule hydrogen bonds (V147, D228).	–	^470^
Compound6	Structure-based optimization (2016)		Y220C Kd = 37.2 μM	Structural stability	X-ray: The CF3 group interacts with L145 and W146, C220.	–	^471^
PK11007	Fragment screening (2016)		Y220C IC50: 2.3 to 42.2 μM	Thermostabilized, structural stability	X-ray: Covalent Modification C182, C277		^477,478^
MB710	Fragment screening (2018)		Y220C Kd = 4 μM	Thermostabilized, structural stability	X-ray: Structural water molecule hydrogen bonds (Val147 and Asp228) and hydrophobic interactions of the N-ethyl group with P151, P152, P153 T155.		^472^
L5	Structure-based desjgn (2018)		Y220C	Zinc ion delivery	Docking: Cavity of p53-Y220C	–	^475^
PK9318	In silico and fragment-based screening (2019)		Y220C Kd = 2.6 μM	Structural stability	X-ray: The secondary amine forms hydrogen bonds with D228. There are interactions the sulfur atom and C220, other hydrophobic interactions: T150, P153, P222, P223.		^473^
L^I^	Structure-based design (2019)		Y220C	p53 aggregates and Zinc ion delivery	Native mass spectrometry: A p53 mutant binds two L^I^ Delivered zinc bound to C238, C242 and C176	–	^499^
L^H^	Structure-based design (2019)		Y220C	Zinc ion delivery	Native mass spectrometry: Does not bind p53 Delivered zinc bound to C238, C242 and C176	–	^499^
PC14586	(2020)	–	Y220C Kd ~ 2.5 nM	Structural stability	-	NCT04585750 Recruiting I/II	^479^
**Targeting p53 aggregation compounds**							
ReAcp53	Sequence-based design (2016)	H-RRRRRRRRRRPILTRITLE-OH	p53 aggregates	Thermostabilized, transcriptional activity	Computational Analysis: p53 (251–258)	–	^490^
ADH-6	Screening an oligopyridylamide library (2021)		p53 aggregates	Thermostabilized, transcriptional activity	NMR: P53-DBD	–	^496^

Peptide and antibody drugs							
Drugs	Discovery method (Report time)	Chemical structure	p53 targets	Rescue potency	Method: Detailed interactions	Identifier/phase/status	Refs
Peptide 46	p53 C-terminus (1997)	GSRAHSSHLKSKKGQST SRHKK	Mutant p53	Restore conformation and transcriptional activity	Pull-Down: P53-DBD and CTD	–	^548^ ^549^
CDB3	Designed based on 53BP2 (2002)	REDEDEIEW-NH2	Structural mutants	Restore conformation and transcriptional activity	NMR: P53-DBD	–	^507,510^
P28	Azurin-derived peptides (2009)	LSTAADMQGVVTDGMASGLDKDYLKPDD	WT and mutant p53	Inhibits p53 degradation	Structural simulations: P53 Loop L1, L7, L8	NCT01975116 Completed I NCT00914914 Completed I	^512^
pCAPs	Phage display technology (2016)	Peptide	Mutant p53	Restore conformation and transcriptional activity	–	–	^511^
Nb139	VIB Nanobody Service Facility (2014)	Antibody	WT and mutant p53	–	X-ray: T32, A34, W54, P101	–	^527^
Nb3	VIB Nanobody Service Facility (2014)	Antibody	R175H, R282W	Inhibits transcriptional activity	X-ray: T32, A34, W54, P101	–	^527^
H2-scDb	Screening and design optimization (2021)	Antibody	R175H Kd = 86 nM	T-cell immune response kills mutated p53 cells	X-ray: 175H	–	^530^

*WT* wild-type

**Table 3:** Scope of rescue drugs for mutant p53

Drugs	p53-DNA contact	Restore the p53 structure	Refs
PRIMA/APR-246	R248Q, R273H	R175H	^386,410^
ATO	–	Structural mutants: R175H, G245S, R249S, R282W and so on.	^431^
PAT	–	Temperature-sensitive mutants: V272M, P278A, Q136P and so on.	^432^
ZMC1	–	R175H	^441^
UCI-LC0023	–	R175H	^462^
PhiKan083 PhiKan5196 PK7088 (PK7242) Compound9 Compound6 PK11007 MB710 L5 PK9318 LI LH PC14586	–	Y220C	^467–478^
ReAcp53	R248Q	R175H	^490^
ADH-6	R248W	–	^496^
Peptide 46	R273H	–	^549^
CDB3	R273H	R175H, G245S, R249S	^507,510^
pCAPs	R273H	R175H, R280H and so on.	^511^

##### PRIMA-1 and APR-246

PRIMA-1 and APR-246 are prodrugs that are converted into the bioactive compound methylene quinuclidinone (MQ). PRIMA-1 was obtained by screening a library of compounds.^386^ PRIMA-1 restores the wild-type conformation and DNA contact of p53 mutants, and induces transcription of the downstream target genes BAX, P21 and PUMA.^386–388^ After PRIMA-1 treatment of SKOV-His-175 cells, a 46% increase in folded p53 protein was observed using the p53 conformation-specific monoclonal antibody PAb1620.^386^ Further studies revealed that APR-246 (PRIMA-1^Met^, eprenetapopt), a methylated derivative of PRIMA-1,^389^ has stronger antitumor activity and fewer toxic side effects than PRIMA-1, and inhibits the growth of p53 mutant tumors of various origins in combination with other anticancer drugs.^390–396^ Tumor cell lines carrying *TP53*, *TP53* mutations, or *TP53* deletions responded to APR-246 treatment.^397–400^ However, growing evidence suggests that APR-246 exerts its effects through a multitude of pathways independent of p53,^401–404^ such as downregulating glutathione concentrations in tumor cells,^405,406^ regulating oxidation-reduction homeostasis^407–409^ in tumor cells to trigger ferroptosis,^410–412^ and inducing endoplasmic reticulum stress^413^ or unfolded protein responses.^414^

Clinical and preclinical data showed that the combination of APR-246 and azacytidine (AZA) showed synergistic activity in patients with myelodysplastic syndromes (MDS), AML and solid tumors carrying *TP53* mutations with an acceptable safety and tolerability profile.^415–420^ Food and Drug Administration and European Medicines Agency have granted APR-246 orphan drug status and FTD for the treatment of MDS carrying the *TP53* mutation. Aprea Therapeutics recently announced preliminary data from its Phase III clinical trial. In a cohort of 154 intention-to-treat patients, the complete remission rate for APR-246 in combination with AZA was 33.3%, compared to 22.4% in the AZA alone group. Although the complete remission rate was higher with the combination, it did not reach statistical significance (https://www.aprea.com). Data from another clinical study could explain this phenomenon. Decitabine alone (a hypomethylating agent, similar to AZA) was able to produce a very high response rate (100%) in patients with AML or MDS carrying a *TP53* mutation.^421^ This suggests that the therapeutic effect of the hypomethylating agent may be dominant in myeloid malignancies carrying *TP53* mutations.

**Fig. 9:**
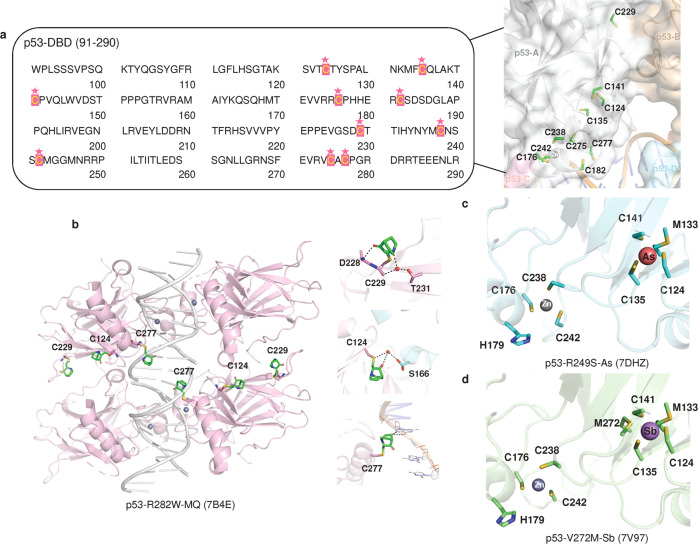
Compounds targeting cysteine in p53-DBD. **a** The amino acid sequence and structure of p53-DBD, cysteine is highlighted and labeled, p53 tetramers are labeled as (**a**–**d**), respectively and cysteines are shown as sticks. **b** MQ bound to C124, C229 and C277 in p53-R282W-DNA tetramers. The MQ conjugates are in stick representation (green). **c** Structure of p53-bound arsenic and zinc ions. **d** Structure of p53-bound antimony and zinc ions. The interacting amino acid residues are shown as sticks

MQ can covalently bind to cysteine to restore the function and conformation of mutant p53. MQ is a very active Michael receptor that preferentially and reversibly binds to the soft nucleophile cysteine thiol of p53.^422^ Theoretically, all cysteines exposed on the surface of p53 are potential modification sites of Michael addition reaction^285,423^ (Fig. 9a). Computational docking indicated that Cys124, located in the center of the L1/S3 pocket of the p53 core region, may be the site of the MQ modification.^424^ In 2021, Degtjarik and colleagues investigated the mechanism of MQ reactivation of mutant p53 based on structure.^425^ Based on the DNA contact surface mutant R273H/R273C and the structural mutant R282W, six cysteines, Cys124, Cys182, Cys229, Cys273, Cys275 and Cys277, were identified to bind to MQ. Among them, the formation of new hydrogen bonds between MQ-Cys277 and DNA stabilizes the protein‒DNA interface, while MQ-Cys124 and MQ-Cys229 stabilize the local conformation and support the p53 dimer interface (Fig. 9b). MQ-Cys182, MQ-Cys275 and MQ-Cys273 are only observed in the structures absent from DNA, and seem to be incompatible with DNA binding. These conjugates form intramolecular interactions or intermolecular interactions with neighboring p53 molecules, stabilizing a p53 dimer different from that in p53-DNA tetrameric complexes. Therefore, MQ shows great diversity in reacting with p53 cysteines, while it worth considering whether each conjugate is beneficial for p53 rescue. Theoretically, MQ can bind to any exposed cysteine and GSH can also bind covalently to MQ,^411^ which may be one of the reasons for the antitumor activity of MQ.

##### ATO

Arsenic trioxide (ATO) is a traditional Chinese medicine that leads to complete remission in patients with acute promyelocytic leukemia (APL).^426^ The specific fusion protein PML-RARα is present in more than 98% of APL patients. One of the mechanisms by which ATO treats APL is its ability to bind directly to the cysteine in the zinc finger structure of the fusion protein, mediating the ubiquitinated degradation of PML-RARα.^427^ Subsequently, a series of reports showed that ATO induced apoptosis in tumor cells carrying *TP53* mutations.^428–430^

Lu Min’s team found that ATO could help stabilize the structural mutation of p53 (R175H) and restore the cancer suppressive activity of p53.^431^ The crystal structure showed that the DNA-binding domains of Cys124, Cys135, Cys141 and Met133 formed an As-binding pocket, with direct interaction of arsenic with Cys124, Cys135 and Cys141 and van der Waals forces between the side chain of Met133 and the arsenic atom (Fig. 9c). Most structural p53 mutants were rescued to varying degrees by ATO, but mutations in the DNA contact surface (R248Q, R273H) were not observed. ATO increased the thermostability of structural mutant p53 by 0.9–6.5 °C, restored the protein folding state close to wild-type, and increased transcriptional activity by several fold to 44-fold. Studies on hematologic tumors showed that ATO significantly prolonged the median survival time in mice; and studies on solid tumors showed that ATO-treated mice had only 10–20% of the tumor volume of the control group.^431^

##### Potassium antimony tartrate (PAT)

Subsequently, the Lu Min’s team screened compounds for temperature-sensitive p53 mutant proteins, using thermostability as a criterion for rescue. Potassium antimony tartrate (PAT) was identified as binding to the p53 V272M mutant (Kd = 9.09 μM) in a non-covalent manner, increasing its thermostability by 3.6 °C and remarkably restoring protein folding and transcriptional activity.^432^ PAT is an antiparasitic agent that has been used as a treatment for leishmaniasis,^433,434^ and has since been found to have antitumor activity.^435,436^ PAT is similar to ATO in that both can induce apoptosis in APL cells.^437,438^ PAT and ATO bind to mutant p53 at similar sites (Fig. 9d), but differ in two aspects, with PAT non-covalently binding mutant p53 and preferentially rescuing temperature-sensitive mutants (a subtype of structural mutants), whereas ATO covalently binding mutant p53 and apparently rescuing more structural p53 mutants.^432^ Notably, the ATO and PAT studies comprehensively and experimentally compared the set of representative mutant p53-rescuing compounds. PAT exhibited specific p53 V272M antitumor activity in both cellular and xenograft mouse models. Further testing identified 65 of p53 mutants that could be rescued by PAT, but all were non-hotspot mutations.^432^ A clinical trial of antimony for the treatment of MDS/AML with *TP53* mutations is currently underway.

##### ZMC1

Zn^2+^ is crucial for DNA recognition by p53. Zn^2+^ can help stabilize the interaction between p53 and DNA by tetrahedral coordination with the imidazole group of H179 and the thiol groups of Cys238, Cys242 and Cys176^298^ (Fig. 9c, d). Conversely, the lack of Zn^2+^ affects the correct folding and DNA recognition of the p53-DBD protein.^439^ Therefore, helping zinc ions to bind in the correct position may be an effective strategy to target p53.

R175H is one of the p53 hotspot mutations and is classified as a Zn^2+^-binding mutation. The binding affinity of DBD-R175H to Zn^2+^ was significantly reduced compared to that of wild type.^440,441^ On the basis of this hotspot mutation, NSC319726, also known as ZMC1, was identified from 48129 compounds.^441^ ZMC1 helped coordinate zinc ions at appropriate sites to restore the structure and function of the R175 mutant^442^ and significantly inhibited tumor growth in mice carrying the R175H mutation.^441^ ZMCI treatment increased the folded R175 mutant protein by twofold.^442^ ZMC1 acts as an ionophore,^443^ transferring zinc ions from the extracellular space to the cytoplasm, maintaining the cytoplasmic zinc ion concentration within an appropriate range and thus reactivating the p53 mutant.^444–446^ In addition, ZMC1 can chelate redox-active copper,^447^ increase intracellular reactive oxygen levels and decrease glutathione concentrations to exert antitumor effects.^441,444^

ZMC1 acts as an ionophore,^443^ transferring zinc ions from the extracellular space to the cytoplasm, maintaining the cytoplasmic zinc ion concentration within an appropriate range and thus reactivating the p53 mutant.^444–446^ In addition, ZMC1 can chelate redox-active copper,^447^ increase intracellular reactive oxygen levels and decrease glutathione concentrations to exert antitumor effects.^441,444^

##### Other compounds

Salim et al. identified a thiosemicarbazideb compound COTI-2 that showed inhibitory activity in a variety of tumors by using a computational platform.^448^ COTI-2 inhibits tumor growth in a p53-dependent and p53-independent manner, ultimately leading to cell cycle arrest and apoptosis.^448–450^ COTI-2 was reported to restore DNA-binding properties to the p53-mutant protein,^449,451–453^ whereas it is obscure whether COTI-2 can physically bind p53. COTI-2 is currently in phase I clinical trials for the treatment of gynecologic malignancies or head and neck squamous cell carcinomas.

There are also small molecule drugs that have not been structurally studied but may interact with the cysteine residues of p53-DBD and restore the wild-type conformational and transcriptional function of p53. CP-31398 was screened from a library of >100,000 compounds originally identified to rescue the mutant p53.^454–456^ However, subsequent studies have shown that CP-31398 does not interact with p53-DBD or full-length p53, but rather acts as an intercalator and may interact with p53 during biosynthesis. CP-31398 inhibited ubiquitination and degradation of p53^457^ and activated BAX independently of p53 to promote apoptosis of tumor cells.^455^ Molecular docking structures revealed that STIMA-1,^458^ MIRA-1^459–461^ stictic acid^424^ and UCI-LC0023^462^ could bind in the L1/S3 pocket of p53-DBD, probably to the thiol group of Cys124 in this transiently opened pocket.^424^

Some of these broad-spectrum mutant p53 rescue compounds have been reported to bind p53-DBD cysteine, helping restore the correct folding and thermostability of mutant p53. Complex structures show that MQ can bind to six of the ten cysteines,^425^ while ATO^431^ and PAT^432^ bind at a pocket formed by Cys124, Cys135, Cys141 and Met133. p53 has ten cysteines in its DBD (Fig. 9a). Theoretically, these cysteines are all potential sites for modification. Amino acids exposed on the surface of p53-DBD, Cys182, Cys229 and Cys277, are the most susceptible sites for modification. Cys182 and Cys229 are located on the p53 dimer contact surface and modification of these two sites may cause non-functional artificial dimers.^463^ Cys277 is located on the DNA contact surface and modification of this site may result in spatial site blocking that inhibits p53 binding to DNA.^463^ Cys176, Cys238 and Cys242 are relatively unlikely to be modified, as they involve the coordination of a zinc atom.^298^ Besides, Cys124, Cys135, Cys141 and Cys275 are located in the p53-DBD core region, which is exposed when the protein is not fully folded or conformationally altered, and may also be the site of modification. Specific targeting of these sites may be more helpful in restoring the structure and function of mutant p53.

#### Allele-specific mutant p53 rescue compounds

##### Y220C

**Fig. 10:**
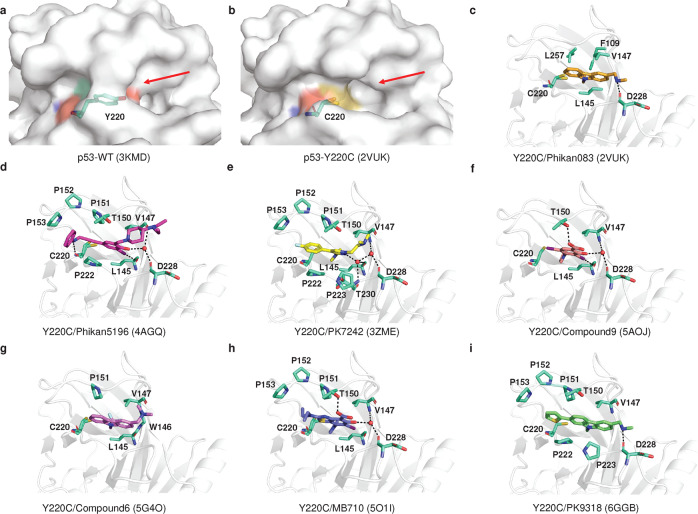
Structure of the p53 mutant Y220C with small molecules. **a** p53-WT surface (PDB: 3KMD). **b** p53-Y220C surface (PDB: 2VUK). **c** p53-Y220C/Phikan083 (PDB: 2VUK). **d** p53-Y220C/Phikan5196 (PDB: 4AGQ). **e** p53-Y220C/PK7242 (PDB: 3ZME). **f** p53-Y220C/Compound9 (PDB: 5AOJ). **g** p53-Y220C/Compound6 (PDB: 5G4O). **h** p53-Y220C/MB710 (PDB: 5O1I). **i** p53-Y220C/PK9318 (PDB: 6GGB). Water molecules are red spheres, and hydrogen bonds are black lines. The interacting amino acid residues are shown as sticks (colored greencyan)

The Y220C mutation in p53 is a relatively specific mutation. Y220 is far from the site where p53 binds to DNA, while the tyrosine mutation between S7/S8 becomes cysteine, forming a hydrophobic cavity on the p53 surface.^464,465^ Therefore, targeting the hydrophobic pocket of Y220C becomes an ideal drug target, helping p53 to restore normal folding without interfering with p53 binding to DNA.^466^ Several small molecules have been shown to bind to the hydrophobic pocket of Y220C and restore p53 function^467–478^ (Tables 2–3, Fig. 10a–i).

C14586 is the first orally bioavailable mutant p53 protein-selective reactivator that selectively binds to the cleft produced by the p53 Y220C mutant protein, thereby restoring wild-type p53 protein structure and tumor suppressor function.^479^ Preclinical trials have shown that continuous oral administration leads to complete tumor regression in 80% of mice.^479^ An ongoing clinical phase 1/2 study will evaluate the safety, tolerability and antitumor activity of C14586 in adult patients with advanced or metastatic solid tumors with the Y220C mutation.^480^

The hot spot mutation Y220C is a particularly suitable target site for structure-based drug design. The mutant p53 temperature sensitivity can be repaired by small molecule drugs, and the cavity that appears due to the mutation is also a good target for small molecules. Structure-based drug design and optimization may lead to new strategies for patients with tumors carrying the p53-Y220C mutation.

#### Targeting p53 aggregation

**Fig. 11:**
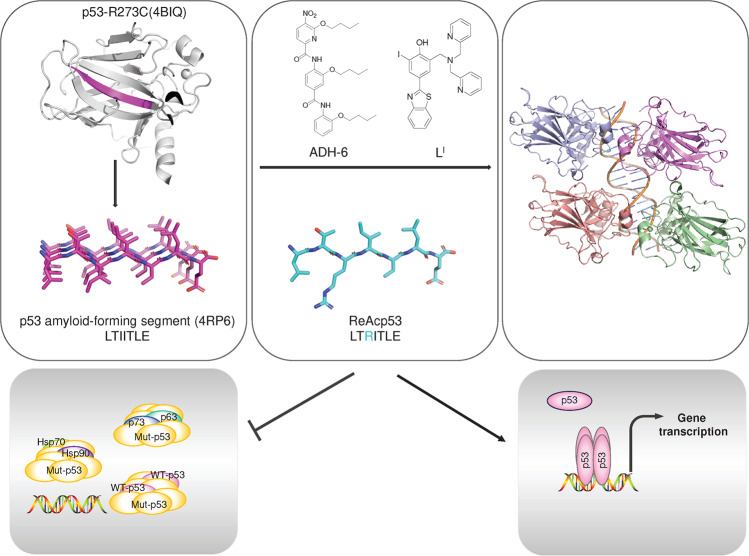
Targeting p53 aggregation. p53-DBD has an amyloid-forming segment, LTIITLE, that forms Mutant p53 aggregates. ReAcp53, ADH-6 and L^I^ inhibit p53 aggregation and restore the conformation and function of p53

p53 is a temperature-sensitive protein. Once mutated, its structural stability is compromised, exposing adhesion sequences encased in the hydrophobic core of p53, which drives the formation of p53 aggregates,^481^ thereby depriving p53 of its DNA recognition function and proapoptotic capacity^482,483^ (Tables 2–3, Fig. 11). Amyloid aggregates caused by *TP53* mutations have been found in biopsy specimens from ovarian and breast cancers.^484,485^

##### ReACp53

The formation of p53 aggregates is considered to be an amyloid lesion, where two β-sheet layers stack in parallel to form a tight complementary spatial zipper, forming amyloid fibrils mainly through hydrogen bonding interactions between the main and side chains of the β-sheet layer.^486,487^ Several research groups have reported similar sequence positions as highly adherent fragments of p53 (251–258, ILTIITLE) (Fig. 11). p53 mutations result in reduced p53 stability and expose the highly adherent fragment that drives p53 aggregation, which is effectively inhibited by the I254R mutation.^457,488,489^ Soragni et al. designed a cell-penetrating peptide (named ReACp53) that inhibits amyloid aggregation of the mutant p53 protein and restores the transcriptional function of p53 and its ability to induce mitochondrial apoptosis.^490^ ReACp53 treatment increased the nuclear localization of mutant p53 by 70–100% and approximately doubled the transcriptional activity.^490^

Molecular dynamics simulations were used to study the binding characteristics of the ReACp53 peptide to the R175H mutant p53-DBD.^491^ The results show that ReACp53 has hydrophobic interactions with residues Leu188 and Leu201 and forms salt bridges or hydrogen bonds with residues Asp186, Glu198, Asp204, Glu 221 and Glu 224, which prevent the aggregation-prone region (residues 182–213) from being exposed. In addition, the complex formed by the ReACp53 peptide and the R175H mutant p53-DBD has the same positive net charge as wild-type p53. The aggregation of p53 was inhibited by electrostatic repulsion.^491^ ReACp53 also inhibits the growth of various tumors characterized by p53 mutations.^490,492–494^

ReACp53 inhibits the aggregation of mutant p53 to rescue p53 function, but it is clear that its non-p53 targets are also present in cancer cells. In p53-silenced cells, the response of ReACp53 was not abolished, and addition of this peptide to p53-silenced two-dimensional cultured cancer cells resulted in rapid apoptosis.^495^ Thus, although ReACp53 was designed as a mutant p53 rescue compound, its exact mechanism of action and targeting in tumors and whether it acts primarily by targeting p53 need to be further elucidated.

##### ADH-6

ADH-6, Kd = 366 nM, is a cationic tripyridylamide obtained by screening the oligopyridylamide library.^496^ Previous studies have reported that these α-helix-like mimics can mimic the secondary structure of proteins and effectively regulate amyloid aggregation^497,498^ (Fig. 11). Using NMR spectroscopy, ADH-6 was shown to bind not only p53-prone aggregation sites but also multiple regions of p53-DBD, including sheet 1, 3, 4, 6, 7 and helix 2. Subsequently, researchers also found that ADH-6 dissociated p53 mutant aggregates and selectively induced apoptosis in multiple p53 aggregation-prone mutated cancer cells (R248W, R248Q, R175H, R273H, Y220C and R280K). In addition, the unfolded state of mutant p53 was reduced by 24–50% after ADH-6 treatment of cells, and the expression of downstream target genes p21, Noxa and BAX was significantly increased.^496^

#### L^I^/L^H^

Miller et al. designed two bifunctional ligands (L^I/^L^H^) based on other compounds that bind amyloid proteins.^499^ L^I^ and L^H^ have the same structural features except for an iodine substituent at the ortho position. The iodine atoms may form halogen bonds that bind to exposed hydrophobic amino acid residues and regulate the aggregation of p53 (Fig. 11). L^I^ and L^H^ acted as metallochaperones binding to zinc ions, which increased the intracellular zinc ion level and improved the binding ability of zinc ions to mutant p53, thus regulating mutant p53 aggregation. L^I^ and L^H^ also increased the expression of Noxa and p21 by 1.5 to 4.3-fold. Meanwhile, transmission electron microscopy observation confirmed that mutant p53 could significantly inhibit the formation of aggregates when cocultured with L^I^.^499^

##### HDAC6/Hsp90 inhibitors

Mutant p53 aggregates not only induced wild-type p53 aggregates but also coaggregated with p63 and p73^495,500^ (Fig. 11). The p53 aggregates led to upregulation of Hsp70 and Hsp90^98^ (Fig. 11). Hsp70 inhibited MDM2-mediated degradation of mutant p53 ubiquitination and led to transient exposure of p53 adhesion sequences, increasing the formation of p53 aggregates.^501^ The interaction between Hsp90 and mutant p53 prevented the ubiquitinated degradation of mutant p53 protein.^502^ Thus, disruption of the HDAC6/Hsp90 complex by HDAC inhibitors^503^ or Hsp90 inhibitors^504^ induces degradation of the p53 mutant.

#### Peptide and antibody drugs targeting protein interactions

Peptide drugs and antibody drugs are characterized by high specificity and good safety profiles. With the maturation of biotechnology, an increasing number of peptide and antibody therapeutics are beginning to emerge that can bind to mutated p53 and restore the function of the p53 mutant (Tables 2–3). However, although peptide drugs can enter cells to restore p53 function, it is difficult to maintain stability and function after entering the human body.^505,506^

##### CDB3

A nine amino acid peptide, CDB3, was designed based on the p53 binding protein 53BP2 region (490–498).^507^ The NMR structure showed that CDB3 interacts with multiple sites in the DBD region, concentrated between loop 1, helix 2 and sheet 8, located at the edge of the DNA-binding site and partially overlapping with the DNA-binding site.^507^ Further experiments revealed that CDB3 could bind to G245S and R249S structural mutants,^508^ restore conformational folding,^509^ improve the affinity of the β-sheet I195T mutant to DNA, and restore the transcriptional activation function of p53 (R175H, R237H). Upon binding of CDB3 to mutant p53, the p53 protein is activated, and target genes compete for the position of binding CDB3, which in turn is released back into the cell and continues to target other mutants.^507,510^

##### pCAPs

A series of p53 conformation-activating peptides, or pCAPs, were identified using phage display technology. Despite the absence of clear structural information, pCAPs were shown to bind to misfolded p53 mutants, forcing p53 conformational recovery and activating transcription of p53 downstream target genes. Significantly reduced mouse tumor size in mouse xenograft models of breast, ovarian and colon cancer.^511^

##### p28

The amphipathic penetrating peptide p28 contains twenty-eight amino acids from Pseudomonas aeruginosa.^512–514^ It preferentially enters cancer cells and inhibits their proliferation by stabilizing the expression of p53.^515^ p28 forms a complex with the DBD of p53, and structural simulations show that the main binding sites of p28 are in the nonmutagenic loop L1 (amino acids 112–124) and the mutagenic loop L7/L8 regions (Y220C, P223L).^516^ p28 inhibits the interaction between the E3 ligase COP1 and p53,^517^ improves the posttranslational stability of p53, and increases p53 protein expression in various p53 wild-type or mutated tumor cell lines.^518,519^ These results provide foundational evidence that p28 performs well in phase 1 clinical trials in patients with various p53 wild-type or mutant tumors.^520–522^

##### Nb3 and Nb139

**Fig. 12:**
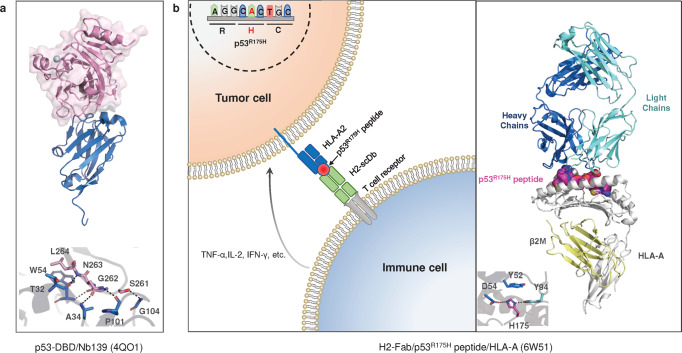
Antibody drugs against p53. **a** Structure of the Nb139/p53-DBD complex. p53-DBD shown as cartoon/surface (colored pink). Nb139 shown as cartoon (colored marine). **b** Mechanism of action and structure of the bispecific antibody H2-scDb. H2-scDb is a bispecific antibody that recognizes the p53^R175H^ mutant peptide presented on the cell surface by HLA-A and binds to the T-cell receptor, activating T cells and releasing cytokines to kill tumor cells. The interacting amino acid residues are shown as sticks. Hydrogen bonds as black lines

Nanobodies containing only a heavy chain variable region (VHH) but still retain antigen specificity and high affinity^523^ have proven their value in cancer therapy and diagnosis in recent years.^524–526^ Two p53-binding nanobodies, Nb3 and Nb139, were obtained by immunization and panning procedures.^527^ Nb3 binds to the “structural mutations” R175H and R282W, and Nb139 binds to both wild-type and mutant p53, inhibiting the transcriptional capacity of p53. The cocrystal structure shows that the three complementary determining regions (CDRs) of Nb139 interact with p53-DBD by forming backbone or side chain hydrogen bonds and van der Waals forces (Fig. 12a). Compared with the wild-type p53 structure, the Nb139/p53-DBD complex maintains the structural and DNA-binding properties of p53, which provides an innovative approach to study p53.^527^

##### H2-scDb

Mutant p53 is hydrolyzed intracellularly to produce a peptide known as a mutation-associated neoantigen that can be presented on the cell surface after forming a complex (pHLA) with human leukocyte antigen (HLA) proteins.^528,529^ In two patients with metastatic epithelial carcinoma carrying the p53^R175H^ mutation, Malekzadeh et al. identified the neoantigen as the HMTEVVRHC peptide, which can be recognized and formed into a complex by HLA*02:01^530^ (Fig. 12b). To exploit this tumor-specific surface antigen, the researchers screened the antibody fragment library and optimized the design to identify a TCR-mimetic antibody in the form of a single-chain double antibody (scDb) with Kd = 86 nM.^529^ The structure revealed that the p53^R175H^ peptide occupies the binding cleft between α1 and α2 of HLA-A*02:01. The His175 amino acid residue in the p53^R175H^ peptide fragment plays a significant role in direct contact with H2-Fab. The imidazole side chain of His175 forms hydrogen bonds with Asp54 (CDR-H2) and Tyr94 (CDR-L3) and forms a π-π stacking interaction with Tyr52 (CDR-H2) (Fig. 12b). Functionally, H2-scDb bound specifically to the p53^R175H^/HLA complex and efficiently induced T-cell immune responses. In multiple myeloma xenograft mice carrying p53^R175H^, H2-scDb effectively regressed tumors.^529^

#### Second-site suppressor mutation

Mutant p53 usually affects the binding of p53 to DNA or the conformation of p53. Second site suppressor mutations could help correct the conformation of mutant p53, improve the stability of p53-DNA binding, and provide a theoretical basis for the reactivation of mutant p53 (Table 4).^531–545^

**Table 4:** Second-site suppressor mutation

Mutation	Second-site suppressor mutation	Mechanism	Reference
G245S	H178Y	Contributes to tetramer formation and restores transcriptional activity.	^533^
	N239Y	Restores p53 stability to wild-type levels and improves DNA binding.	^531,542^
	T123P	Improves structural stability and restores transcriptional activity.	^537^
	S240N	Improves structural stability and restores transcriptional activity.	^537^
R249S	H168R	Restore DNA contact and transcriptional activity.	^531,532^
R249S	T123A + H168R	Improves structural stability and restores transcriptional activity.	^532,537^
V143A	N268D	Stabilized the global folding of p53.	^531,534,537^
V143A	M133L + V203A + N239Y + N268D	Improves thermal and structural stability	^534^
N131Y	N239Y	Restored p53 function and inhibited tumor growth.	^535^
R273C	S240R	Restore protein-DNA interactions.	^536,543^
R273C	T284R	Restore protein-DNA interactions.	^345^
R273H	T284R	Restore protein-DNA interactions.	^536,543^
R273H	N263Y	Improves structural stability.	^540^
R273H	N200Q + D208T	Improves structural stability.	^540^
R273H	N235K + N239Y	Improves structural stability.	^540^
R273H	S240R	Improves structural stability.	^540^
Y220C	A138G	Improves structural stability.	^540^
Y220C	L137R	Improves structural stability.	^540^
R248Q	H115N	Improves structural stability and restores transcriptional activity.	^541^
V157F	N235K + N239Y	Improves thermal and structural stability.	^545^

Crystal structures show that the second-site suppressor mutations usually cause only small local structural changes, whereas do not alter the overall structure of the p53 protein.^534,536,545,546^ These second-site suppressor mutations act through two types of mechanisms: supplying a new DNA-contacting amino acids when rescuing DNA-contacting mutants, or increasing thermostability when rescue structural mutants.^537,547^ These mutants provide the basis for designing drugs that restore the transcriptional inactivation and instability of p53 caused by mutations.

### Other therapies

The TET and CTD have essential roles in the proper folding and functional regulation of p53. There are no drugs that specifically target these two structural domains. A synthetic peptide (361–382) extracted from the p53-CTD region (361–382, peptide 46) can interact with the DBD and CTD regions of p53,^548^ and the addition of different concentrations of peptide 46 restores the DNA binding ability of p53 mutants (R273H, R248W, R175H, R249S, V143A).^549^ The fusion of the p53 protein with the N-terminal spider silk domain contributes to the stability of the p53 protein.^550^ The therapeutic effects and specific mechanisms targeting these two structural domains need further exploration.

Researchers have also developed innovative therapies that target mRNA for protein degradation. For example, a specific deoxyribozyme (DZ-249A) has been designed to target the mutation site of *TP53* to degrade the mRNA of mutant *TP53*, thereby reducing the expression of the mutant p53 protein.^551,552^ Combination therapy of multiple mechanisms is also a promising strategy; for instance, the combination of MDM2 and BCL-2 inhibitors can effectively induce apoptosis.^553,554^ Moreover, gene therapy and immunotherapy are also new options for targeting p53.

### Therapeutic strategy for truncated p53

Nonsense mutations cause proteins to terminate or end translation earlier than expected, generating truncated proteins. A total of 8.19% of *TP53* mutant tumors were detected to carry nonsense mutations and were cleared by the nonsense-mediated mRNA decay (NMD) machinery during transcription^555,556^ (Fig. 1a). Currently, there are two approaches to rescue truncated p53.^557^ One is to restore production of full-length p53 protein by drug-induced read-through of the premature termination codon. For instance, G418 and NB124 can stably promote the expression of full-length p53 protein.^558,559^ The other is the inhibition of NMD.^560,561^ However, NMD is a potent cellular surveillance mechanism^562^ and non-specific inhibition of NMD may do more harm than good.

### Therapeutic strategy for p53 isoforms

Fourteen isoforms of p53 have been identified: p53, p53β, p53γ, Δ40p53, Δ40p53β, Δ40p53γ, Δ133p53, Δ133p53β, Δ133p53γ, Δ160p53, Δ160p53β, Δ160p53γ, Δp53, and p53ψ.^563–568^ It was shown that Δ133p53 activates the anti-apoptotic BCL-2 protein and antagonizes p53-induced apoptosis.^563^ p53Ψ cannot bind to DNA or transcriptionally activate p53 target genes. However, it induces the expression of epithelial-mesenchymal transition markers and enhances the viability and invasiveness of cancer cells.^565^ Overexpression of Δ133p53α facilitates the long-term propagation of primary epithelial cells in vitro.^569^ These evidences suggest that some p53 isoforms are in marked contradiction to the function of wild-type full-length p53, exhibit oncogenic effects, and support cancer cell proliferation and invasion. Therefore, rescue therapy may not be appropriate for these p53 isoforms, and targeted degradation may be a better strategy to remove their oncogenic effects, such as PROTAC,^570^ ATTEC,^571^ AUTAC,^572^ and LYTAC.^573^

## Conclusion

The *TP53* gene is the most frequently mutated gene in humans. In the 40 years since the discovery of p53, new insights have been made into the gene regulatory mechanisms and tumor suppression pathways of p53. We have learned about an elaborate and complex tumor suppression network, but have not yet discovered its accurate and complete picture. Based on the relationship between the structure and function of p53 and cancer development, many anticancer drugs targeting p53 have been developed. However, there are no approved drugs to date.

### Summary points

Among the molecules developed to target p53, two main mechanistic approaches have been implemented. One is to target wild-type p53 and inhibit the p53/MDM2 complex to prevent its degradation. To date, eleven MDM2 inhibitors are in clinical trials. However, long-term administration of an MDM2 inhibitor may induce acquired resistance related to *TP53* mutations and increased MDMX expression.^574,575^ There is still a lack of MDM2/MDMX dual-target or specific MDMX inhibitors. Moreover, the accumulation of p53 in normal tissues may produce greater toxic effects.^353^

The second is to target p53 mutants. Many therapeutic approaches aim to rescue the function of mutated p53 through small molecule compounds. The development of p53 mutant-rescuing drugs has been hampered by false trials that have proven not to be reproducible in other labs or to function by p53-unrelated pathways. Some drugs were initially identified as p53 mutant activators, but it remains puzzling whether they actually rely on p53 for their action. For example, CP-31398 was initially thought to maintain the wild-type conformation of the mutant p53,^454^ but was shown to be unable to bind p53 in a different lab.^455^ Dr. Soragni states on her website that “we don’t really know what the mechanism is for ReACp53 to kill cancer cells” (http://alice.mbi.ucla.edu/reacp53.html). This may be because most experiments to rescue mutant p53 drugs are performed in cells, and the lack of information on the direct interaction of compounds with proteins leads to our incomplete understanding of the mechanism of drug action. Most of the currently reported drugs that activate p53 mutants have p53-independent antitumor effects. It is undeniable that these drugs do act to rescue mutant p53 function, but it may be ambiguous which is more effective, whether it is mutant p53-dependent, mutant p53-independent, or both. The field of targeting mutant p53 remains a shimmering light after the dawn, and we see hope, but there is still a long road of discovery to go.

The availability of structural data on p53 mutants and their complexes with drugs, combined with data on the functional rescue of the mutants, has helped to understand the rescue mechanism and optimize drug design. For example, a special class of p53 structural mutant, Y220C, leads to a hydrophobic cavity in p53 protein structure.^467^ Small molecules are designed to target this cavity to stabilize the protein structure. ATO^431^ and PAT^432^ bind to an allosteric site exposed within the p53 mutant protein and help stabilize the local structure. They are likely to be effective for structural mutants or temperature-sensitive mutants, but not DNA contact mutants. Those mutations at the DNA contact surface tend to benefit from small molecules that have the ability to restore protein-DNA binding. The structures of MQ with p53 mutants R282W, R273H and R273C show that the binding of MQ with Cys277 supports the p53-DNA interface by complementing additional interactions, while MQ interaction modes exhibit large diversity by binding to some other cystines.^425^ In addition, structural analysis illustrates the basis of a TCR-like antibody H2-Fab specifically targeting the p53^R175H^ peptide-HLA interaction.^529^ Nevertheless, structural information about p53 mutants and their complexes is still insufficient, so we need more structural insights to help target p53 mutants for rational drug design.

### Challenges and perspectives

There are significant challenges in the development of drugs targeting p53. The lack of an established mechanism for protein reactivation and the lack of pockets (except in the case of Y220C) are the two main reasons. It is relatively easy to inhibit protein function by simply using compounds to occupy their active sites, but elusive as to how compound binding leads to reactivation of protein function. In addition, resistance to *TP53* mutations, the off-target effects of the drug, and the possible toxic side effects of p53 accumulation in normal tissues, all make p53 difficult to drug. Currently, the structures of full-length p53 in complex with different DNA targets and full-length partner proteins remain unavailable. Certain p53 mutants cannot be expressed, and their structures are not available, which limits structure-based drug design. In recent years, there have been great advances in cryo-electron microscopy, and artificial intelligence. With these technological advances, we have reason to believe that the structural study of p53 will make more progress in the near future, which will provide a structural basis for the development of p53-targeted drugs.

For p53-targeted therapy, several other factors also need to be considered. First, *TP53* mutations are heterogeneous and not all mutations are equal. Hence a one-drug-fits-all approach may not be feasible for targeting *TP53* mutations.^12,576,577^ Therefore, different p53-targeting drugs may be required for different p53 mutants. Second, p53-targeted therapy alone may not be sufficient to treat cancer. Combination therapy, such as simultaneous blockade of the MDM2-p53 pathway and p53-BCL-2 pathway, may have a synthetic lethal mechanism. Third, there are some other new therapeutic directions that may be explored, such as targeting p53 mRNA, targeting disordered structural domains, targeting mutant protein for degradation, and genome editing using CRISPR-Cas9. CRISPR-Cas9 based gene editing technology has been applied to cancer treatment.^578,579^ With the advancement of CRISPR-Cas9 technology, the correction of *TP53* mutations using CRISPR-Cas9 may become an effective cancer treatment option in the future.

For decades, there has been a lack of effective progress in the development of drugs targeting p53, and p53 was once considered to be an undruggable target. As technology advances, many undruggable targets are becoming druggable, such as KRAS.^580,581^ We have reason to believe that drugs targeting p53 will also make progress. Given the prevalence of *TP53* mutations in human cancers, drugs targeting p53 may bring a breakthrough in cancer therapy.
